# Hyaluronic Acid Receptor Stabilin-2 Regulates Erk Phosphorylation and Arterial - Venous Differentiation in Zebrafish

**DOI:** 10.1371/journal.pone.0088614

**Published:** 2014-02-28

**Authors:** Megan S. Rost, Saulius Sumanas

**Affiliations:** 1 Division of Developmental Biology, Cincinnati Children's Hospital Medical Center, Cincinnati, Ohio, United States of America; 2 Cancer and Blood Diseases Institute, Cincinnati Children's Hospital Medical Center/University of Cincinnati, Cincinnati, Ohio, United States of America; Institute of Molecular and Cell Biology, Singapore

## Abstract

The hyaluronic acid receptor for endocytosis Stabilin-2/HARE mediates systemic clearance of multiple glycosaminoglycans from the vascular and lymphatic circulations. In addition, recent in vitro studies indicate that Stab2 can participate in signal transduction by interacting with hyaluronic acid (HA), which results in Erk phosphorylation. However, it is not known whether Stab2 function or HA-Stab2 signaling play any role in embryonic development. Here we show that Stab2 functions in a signal transduction pathway regulating arterial-venous differentiation during zebrafish embryogenesis. Stab2 morpholino knockdown embryos (morphants) display an absence of intersegmental vessels and defects in the axial vessel formation. In addition, Stab2 morphants show defects in arterial-venous differentiation including the expansion of venous marker expression. Simultaneous knockdown of Stabilin-2 and Has2, an HA synthetase, results in a synergistic effect, arguing that HA and Stab2 interact during vasculature formation. Stab2 morphants display reduced Erk phosphorylation in the arterial progenitors, which is a known transducer of VEGF signaling, previously associated with arterial-venous differentiation. In addition, VEGF signaling acts as a negative feedback loop to repress *stab2* expression. These results argue that Stab2 is involved in a novel signaling pathway that plays an important role in regulating Erk phosphorylation and establishing arterial-venous identity.

## Introduction

Vascular endothelial cells establish their arterial-venous identities prior to the initiation of circulation [1], [2]. Understanding the signaling pathways that lead to proper arterial-venous (A-V) differentiation is critical to develop novel treatments for vascular diseases including arterial-venous malformations, hemorrhage and stroke. Despite recent advances, our knowledge of molecular mechanisms that regulate A-V differentiation is still limited. The zebrafish has emerged as an excellent model organism to study vascular development. In the zebrafish, similar to other vertebrates, the cardiovascular system is one of the first to form, and a beating heart and functional circulatory system are established by 24 hpf [3]. During mid-somitogenesis stages, arterial and venous progenitors of the major axial vessels in zebrafish originate bilaterally in distinct locations within the lateral plate mesoderm and migrate to the midline where they coalesce into the major axial vessels, the dorsal aorta (DA) and the posterior cardinal vein (PCV) [1], [4], [5]. Multiple arterial and venous specific markers become preferentially enriched in the DA or the PCV by 24 hpf, prior to the initiation of circulation [6]. These include arterial-specific *ephrin B2A* (*efnb2A*) [6], *aquaporin8a* (*aqp8a*) [7], *gridlock/hey2* (*grl*) [8] and *claudin5b* (*cldn5b*) [9] and venous specific *eph receptor B4a* (*ephb4a*) [6], *fms-related tyrosine kinase 4* (*flt4*) [7], *mannose-receptor C1* (*mrc1*) [10], *stabilin-2* (*stab2*) [10] and *stabilin1-like* (*stab1l*) [11].

Hh, Vegf and Notch signaling pathways have been previously implicated in A-V differentiation and patterning [12], [13]. In the zebrafish, Shh is secreted from the notochord and the floorplate and upregulates VegfA expression in the ventral part of the somites. It has been demonstrated that VegfA plays a direct role in regulating A-V differentiation. VegfA downregulation in zebrafish results in the loss of arterial marker expression [12]. Zebrafish embryos injected with VegfA-specific mRNA isoforms Vegf_121_ or Vegf_165_ display ectopic expression of arterial markers including *efnb2a* in the cardinal vein [12] and loss of venous marker expression. One of the major intracellular effectors activated by Vegf is MAP kinase signaling. Vegf activation is known to result in PLC-γ mediated Erk phosphorylation specifically in the arterial progenitors. Another branch of Vegf signaling, PI3 kinase/AKT pathway, is thought to have an opposite effect, inhibiting Erk phosphorylation [14]–[16]. Treatment of zebrafish embryos with PI3 kinase inhibitors results in increased arterial and reduced venous differentiation [16]. However, it is not clear how the opposing branches of Vegf signaling are regulated to induce Erk phosphorylation exclusively in the arterial progenitors.

Stabilins comprise the family of large transmembrane glycoproteins containing four domains with EGF-like repeats, seven fasciclin-1 domains and an X-link domain [17]. Two major family members, Stab1 and Stab2 are expressed in sinusoidal endothelial cells where they function as scavenger receptors to clear metabolic waste products from the circulation [18]–[20]. Stab2, in particular, binds to and mediates endocytosis of hyaluronic acid (HA), acetylated-LDL, chondroitin sulfate, heparin and dextran sulfate [20]. Mouse Stab2−/− embryos are viable while Stab1−/−Stab2−/− mutants die from severe glomerular fibrosis [21]. Recent studies have shown that Stab2 deficient mice display increased serum levels of HA as well as reduced tumor metastasis, most likely due to the role of HA in the immune system [22]. Recent studies have suggested that in addition to the role of Stab2 as a scavenger receptor, it may also function in a signal transduction pathway. As demonstrated by in vitro studies, Stab2 interaction with HA resulted in the intracellular Tyr-phosphorylation of Stab2, which then caused an increase in Erk-phosphorylation [23]. HA-Stab2 interaction has also been demonstrated to cause NFκB-mediated gene activation in vitro [24]. However, it is currently not known if Stab2 can function in a signal transduction pathway in vivo. Furthermore, a Stab2 role during embryonic development, if any, remains to be elucidated.

We have previously shown that Stab2 exhibits specific expression in vascular endothelial cells during early stages of zebrafish embryogenesis. Its expression is first enriched in the arterial progenitors while by 24 hpf its arterial expression is downregulated and it becomes preferentially localized to the venous endothelial cells [10]. In this study, we investigated Stab2 function during vascular development in zebrafish embryos. Our results demonstrate that Stab2 knockdown embryos exhibit defects in arterial-venous differentiation. We further show that HA and Stab2 genetically interact suggesting that Stab2 can function as a HA receptor in vivo. Stab2 knockdown perturbs Erk-phosphorylation in zebrafish embryos. These results demonstrate Stab2 function in vivo in regulating A-V differentiation.

## Methods

### Zebrafish strains and Staging

Tg(*kdrl*:GFP)^s843^ [5], Tg(*kdrl*:mCherry)^ci5^ [8], *mib* ^ta52b^ [25], [26], and Tg(*fli1a*:GFP) [27] zebrafish lines were used in this study. Embryos were raised at 28.5°C. All embryonic staging was performed using established morphological staging criteria [28]. Developmental delay in morphant embryos was accounted for by allowing morphant embryos to develop until morphological staging criteria matching wild type embryos was observed in morphants.

### Animal Use Ethics

Animal work was performed under the protocol # 0A08058, approved by the Institutional Animal Care and Use Committee, Cincinnati Children's Hospital Medical Center. Animals were sacrificed by immersing embryos and adults in 2% Tricaine before freezing and disposal to minimize suffering. This euthanasia protocol is consistent with the recommendations of the Panel on Euthanasia of the American Veterinary Medical Association.

### Morpholinos

All morpholinos were obtained from GeneTools with sequences GTAATCATCATGCCGTTCCTTCTAG (Stab2 MO1), GAGCCAGAAAGAAGAAACTGAAAGT (Stab2 MO2), and AGCTGACCGCTTTATCACATCTCAT (Has2). All MOs were dissolved in nuclease free water. For Stab2 experiments, a cocktail containing total amounts of 1.25 ng Stab*2* MO1, 2.5 ng Stab2 MO2, and 3.75 ng p53 MO [29] was injected into 1–2 cell stage zebrafish embryos. A lower concentration containing 1.25 ng total Stab2 MO mixture was injected for synergy experiments. For Has2 experiments, a cocktail containing 8 ng Has2 MO and 2.5 ng p53 MO was injected into embryos. For control experiments, 3.75 ng standard control MO (CCTCTTACCTCAGTTACAATTTATA, Gene Tools)+3.75 ng p53 MO, 7.5 ng Stab2 MO1 alone, 2.5 ng Stab2 MO2 alone, or 3.75 ng Stab2 5 ms MO (CTGGAAGCAACGTCATGCTGATAAC)+3.75 ng p53 MO were injected.

### In situ hybridization

Whole mount in situ hybridization was performed as previously described [30]. Antisense RNA probes for the following genes: *kdrl/flk1* [31], *fli1a* [31], *stab2* [10], *esam/esama* [10], *she* [10], *mrc1* [10], *flt4* [31], *grl* [8], and *aqp8a* [7] were synthesized as described. *Cldn5b* (Open Biosystems cat. No EDR1052-97957361 in pExpress-1 vector) and *stab1l* (Open Biosystems cat. No EDR1052-7386647 in pCMV-Sport6.1 vector) cDNAs were digested with SmaI and EcoRV respectively and both transcribed with T7 polymerase. Experiments were performed at least in duplicate to obtain final N values.

### Immunostaining

Control uninjected and Stab2 morphant embryos were fixed in 4% paraformaldehyde (PFA) at the 20 somite stage, dehydrated in ethanol and stored at −20°C. Embryos were rehydrated and blocked in blocking reagent (Roche). Following blocking, embryos were incubated in rabbit polyclonal anti-phospho-p44/42 MAP kinase antibody (Cell Signaling Technologies) overnight, washed, incubated in rabbit anti-Alexa647 (Invitrogen) for four hours, washed, and incubated in rabbit anti-GFP Alexa-488 (Invitrogen) overnight. Embryos were analyzed and imaged using fluorescent microscopy.

### Chemical treatments

For DAPT experiments, uninjected control and Stab2 Morphant embryos were treated in either 100 µM N-[N-(3,5-Difluorophenacetyl)-l-alanyl]-S-phenylglycine t-butyl ester (DAPT) (Sigma) or 1% DMSO beginning at the 50% epiboly stage. Embryos were incubated in the chemical until time of examination (24 hpf). For LY294002 experiments, Embryos were treated in either 20 µM LY294002 (cell signaling technologies) or 1% DMSO beginning at the 12 somite stage and continuing until the 24 hpf stage at which point embryos were fixed for further analysis.

### mRNA for injection

An equal ratio of Vegf_121_ and Vegf_165_ RNA was injected at a final amount of 50 pg into zebrafish embryos at the 1–2 cell stage. RNA was synthesized as previously described [32]. Full length zebrafish *stab2* cDNA was synthesized using gene synthesis (GenScript) and subcloned into a pT3TS vector [33]. During the gene synthesis, cDNA sequence was altered based on the genetic code redundancy to perform codon optimization and to alter MO binding site (Fig. S4). Stab2 mRNA was made using T3 mMessage mMachine kit (Invitrogen) and injected at a dose of 160 pg per embryo in conjunction with Stab2 morpholino mixture.

### Imaging

Embryos were whole mounted in 3% methylcellulose on glass slides. Images were captured using a 10× objective on AxioImager Z1 (Zeiss) compound microscope with Axiocam ICC3 color camera (Zeiss) or MMR grayscale camera (fluorescent images) (Zeiss). Images in multiple focal plans were captured individually and combined using Extended Focus module within Axiovision software (Zeiss).

### Fluorescent intensity calculations

Embryos immunostained with a P-Erk antibody were imaged as described above. For each embryo, a total of five points along the DA were selected at regular intervals using ImageJ software. The intensity values for these five points were averaged to obtain a single intensity value for each embryo. Additionally, three points just anterior to the DA were selected and averaged using ImageJ software to obtain a background intensity value. This background value was subtracted from the DA intensity value resulting in a normalized intensity.

## Results

### Stabilin-2 knockdown affects intersegmental vessel formation

To determine if Stabilin-2 plays a role during zebrafish vascular development, we used two separate translation blocking morpholinos (MOs) [34] to disrupt Stab2 function. General morphology of embryos was undisturbed upon Stab2 knockdown, but embryos displayed impaired blood circulation including blood pooling in the common cardinal vein (data not shown).

**Figure 1:**
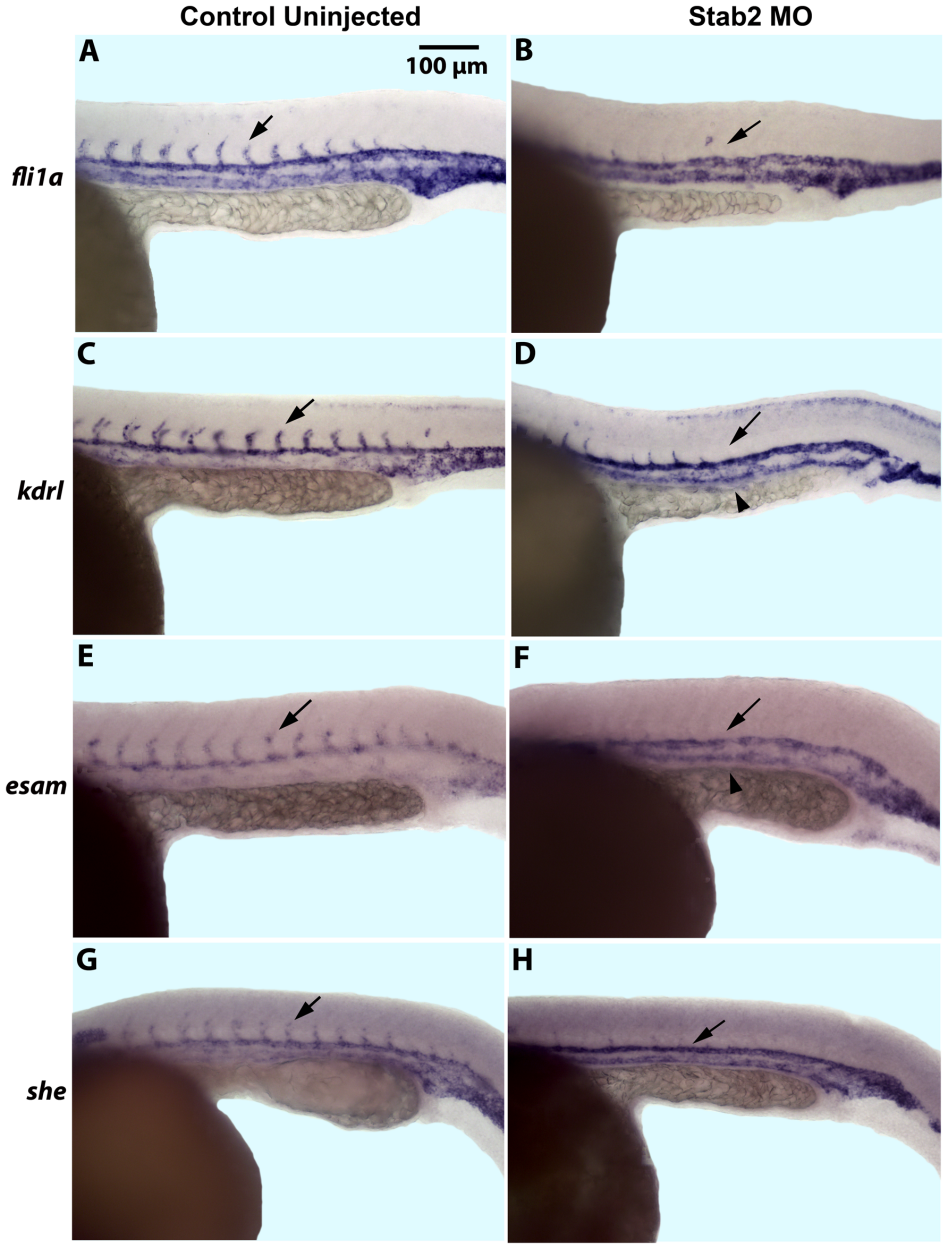
Stab2 morphants display a lack of intersegmental vessels. (A–H) In situ hybridization analysis shows a lack of ISVs in Stab2 morphants as observed by endothelial marker *fli1a* (A and B), *kdrl* (C and D), *esam* (E and F), and *she* (G and H) expression at 24 hpf, as compared to uninjected controls. Arrows indicate ISVs in control embryos and missing ISVs in morphants. Arrowheads indicate stronger PCV expression of *kdrl* and *esam* in morphant embryos (D and F). Lateral view, anterior is to the left; trunk and tail region is shown. Morphants were injected with a cocktail containing Stab2 MO1 and MO2, as well as p53 MO. ISVs: intersegmental vessels; PCV: posterior cardinal vein.

**Figure 2:**
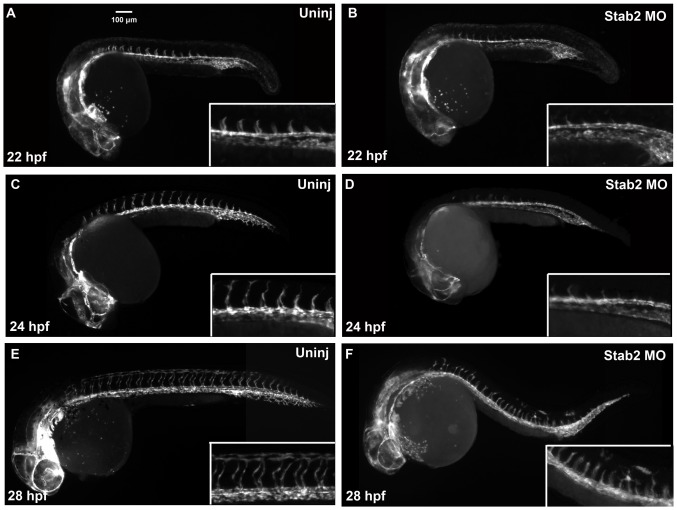
Stab2 morphants display ISV defects at 22–28 hpf. Tg(*kdrl*:GFP) expression in wild type uninjected embryos (A,C, and E) and Stab2 morphant embryos (B, D, and F) at 22 hpf (A,B), 24 hpf (C,D) and 28 hpf (E,F). Morphant embryos display absent (B and D) or reduced (F) ISVs at all three stages. Lateral view shown, anterior is to the left. Morphants were injected with a cocktail containing Stab2 MO1, MO2 and p53 MO.

To analyze vascular development in greater detail, in situ hybridization (ISH) analysis was performed for expression of several different vascular endothelial markers including *fli1a* [31], *kdrl* [31], *esam* [10], and *she* [10] at 24 hpf (Fig. 1). As evident from ISH analysis, Stab2 MO-injected embryos (morphants) displayed either partial or complete absence of intersegmental vessels (ISVs) (Fig. 1A–H, Table S1). This phenotype was observed with each Stab2 MO injected separately or with the mixture of both morpholinos (Fig. S1, Fig. 1). No other morphological defects were observed at these morpholino doses. ISV formation was unaffected by injecting a standard control morpholino at similar concentrations which argues for the specificity of the observed defects in Stab2 morphants (Fig. S1B). To confirm that the observed ISV defects were not an artifact of developmental delay, we observed and imaged Tg(*kdrl*:GFP) embryos from 22 hpf – 28 hpf. ISV sprouts began to form in wt embryos at 22 hpf, while these sprouts were nearly absent from Stab2 morphants (Fig. 2A–B). ISVs were further developed at 24 hpf in wt embryos, but were still nearly absent in Stab2 morphants (Fig. 2C–D). By 28 hpf, significant inhibition of ISV formation was still observed in morphant embryos (Fig. 2E–F).

To further confirm specificity of the observed phenotype, a Stab2 morpholino containing five mismatched base pairs was injected at the 1–2 cell stage (Fig. S2). Transgenic *kdrl*:GFP embryos appeared morphologically normal at 24 hpf with all ISV sprouts intact (Fig. S2A,D). To confirm that Stab2 morpholinos inhibit translation of Stab2 protein, a reporter construct was made by fusing 1 kb Stab2 5′UTR and promoter sequence that included MO binding sites to GFP coding sequence (Fig. S3A). The construct was injected into wild type zebrafish embryos at 1–2 cell stages. These embryos displayed mosaic GFP expression at the 50% epiboly stage (Fig. S3B) while embryos co-injected with the GFP construct and either Stab2 MO1 or MO2 did not display GFP expression anywhere in the embryo (Fig. S3C–D). Together, these experiments show that the morpholinos are effective in blocking Stab2 translation and result in a specific phenotype.

### Stabilin-2 knockdown affects arterial-venous differentiation

**Figure 3:**
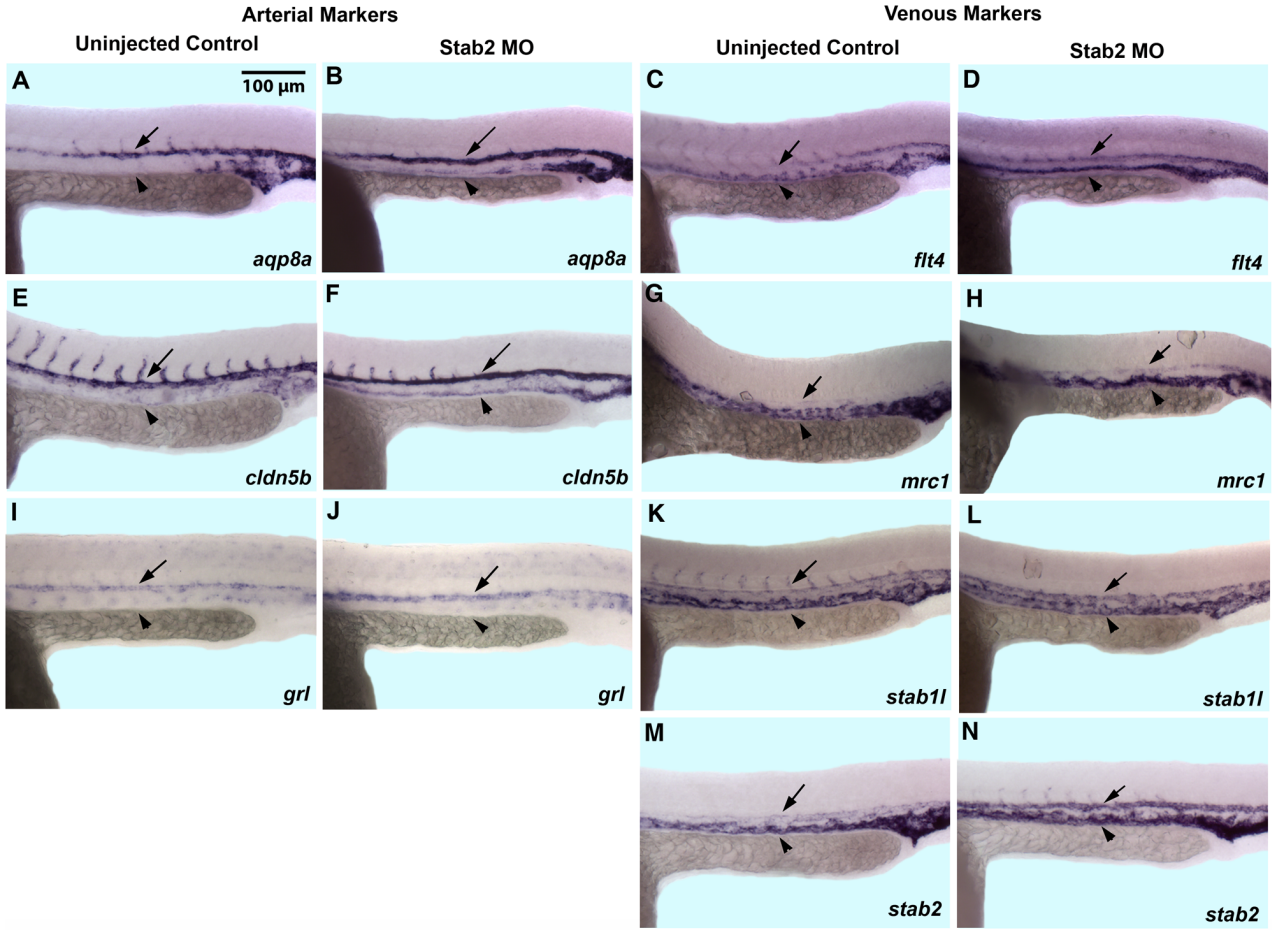
Stab2 morphants display expanded expression of certain arterial and venous markers. (A,B and E,F) As analyzed by in situ hybridization, *aqp8a* (A,B) and *cldn5b* (E,F) expression is restricted to the DA in wild type uninjected embryos while it is expanded into the PCV in Stab2 morphants at 24 hpf. (I,J) Arterial *grl* expression is not affected in Stab2 morphants. (C,D, G,H, and K,L) Expression of venous specific markers *flt4* (C,D), *mrc1* (G,H), and *stab1l* (K,L) is expanded into the area of the DA in Stab2 morphants. (M,N) Venous expression of *stab2* itself is also expanded into the area of the DA at 24 hpf in Stab2 morphants. Arrows indicate DA and arrowheads indicate PCV. Lateral view, anterior is to the left, trunk and tail region is shown. DA: dorsal aorta; PCV: posterior cardinal vein. All embryos are 24 hpf. Morphants were injected with a cocktail containing Stab2 MO1, MO2 and p53 MO.

Inhibition of ISV sprouting observed in Stab2 morphants may be due to the specific Stab2 role in angiogenesis. Alternatively, Stab2 may be involved in an earlier vascular patterning event that precedes ISV sprouting. Because *stab2* displays a dynamic expression pattern and is first observed in the arterial precursors, while subsequently becomes enriched in the PCV by 24 hpf [10], we examined a possibility that Stab2 may function in regulating A-V differentiation. While vascular markers *kdrl* [31] and *esam* [10] displayed higher arterial than venous expression in uninjected control embryos, venous expression of these markers was greatly increased in Stab2 morphants (Fig. 1C–F). Arterial specific markers *aqp8a* [7] and *cldn5b* were expressed in both the DA and PCV at 24 hpf in a fraction of Stab2 morphants (Fig. 3B,F, Table S2) while distinctive DA specific expression was seen in wild type uninjected embryos (Fig. 3A,E). In contrast, arterial specific *hey2/grl* [8] expression was not changed in Stab2 morphants as compared to wild type embryos (Fig. 3I,J, Table S2). The venous specific markers, *flt4* [31], *mrc1* [10], and *stab1l* all showed expression in both the DA and PCV in Stab2 morphants (Fig. 3D,H,L, Table S2) as compared to wild type controls expressing these markers more prominently in the PCV (Fig. 3C,G,K). Expression of *stab2* itself was also expanded into the DA in Stab2 morphants while it was largely restricted to the PCV in control embryos at 24 hpf (Fig. 3M,N, Table S2).

Arterial-venous differentiation defects were specific to Stab2 MO injection because embryos injected with a Stab2 5 base pair mismatch morpholino did not show any apparent expansion of arterial or venous markers when analyzed by ISH with arterial marker *cldn5a* (Fig. S2B,E) or venous marker *stab1l* (Fig. S2C,F). To further demonstrate specificity of the observed phenotypes we attempted their rescue with synthetic zebrafish Stab2 mRNA. Stab2 cDNA sequence was altered based on the genetic code redundancy (Fig. S4) to optimize codon usage for efficient translation and to avoid binding to Stab2 MOs. Stab2 mRNA overexpression did not cause any apparent morphological or vascular defects (data not shown). Coinjection of Stab2 mRNA together with Stab2 MO resulted in a partial rescue of venous marker *flt4* expression, compared to Stab2 MO injection alone (Fig S2G–I, Table S3). Sprouting defects of ISVs were not rescued in this experiment (data not shown), possibly because it is difficult to recapitulate precise endogenous Stab2 expression levels in this experiment and even minor defects in arterial-venous differentiation can result in defective sprouting of intersegmental vessels. These results confirm specificity of the observed defects in Stab2 morphants. They also show that expression of some arterial markers is expanded into the PCV in Stab2 morphants while expression of multiple venous markers is expanded into the DA, which argues for the role of Stab2 in regulating arterial and venous cell identity.

### Stab2 knockdown disrupts normal Erk phosphorylation pattern

**Figure 4:**
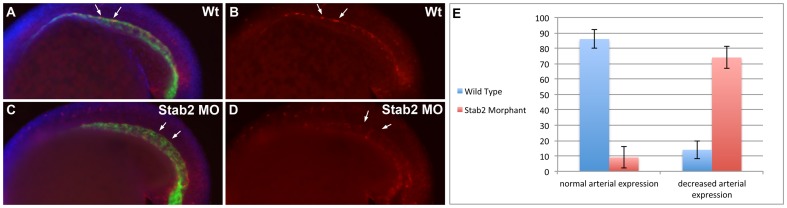
Stab2 knockdown affects Erk phosphorylation. (A–D) Phosphorylated Erk expression is observed in the DA of uninjected control embryos by whole mount immunostaining (A,B), while a majority of Stab2 morphants display a reduction in arterial P-Erk expression (C,D). (E) Percentages of embryos displaying normal or decreased arterial P-Erk expression. Arrows indicate P-Erk expression in the DA (A,C). Experiments performed in a Tg(*fli1a*:GFP) line. Green staining: GFP, Blue Staining: DAPI, Red staining: P-Erk. Lateral view, anterior is to the left. All embryos are at the 20-somite stage. Morphants were injected with a cocktail containing Stab2 MO1, MO2 and p53 MO.

Erk phosphorylation has been previously associated with A-V differentiation [15], [35]. Because Stab2/HARE has been previously implicated in Erk phosphorylation induction in vitro [23], we examined the effects of Stab2 knockdown on Erk phosphorylation in a vascular endothelial specific *fli1a*:GFP reporter line. Stab2 morphants were fixed at the 20-somite stage for this analysis, a time point when phosphorylated Erk expression can normally be seen in the DA progenitors (Fig. 4A,B), and subjected to whole-mount immunostaining with phospho-Erk and GFP antibodies. We found that seventy-three percent of Stab2 morphants display a reduction in phosphorylated-Erk staining in the DA in contrast to fourteen percent of control uninjected embryos (Fig. 4C–E, Table S4). Quantification of fluorescence intensity (see Methods) revealed that Stab2 morphants (n = 13) displayed 2.5-fold reduction (p = 0.0006) in the arterial P-Erk expression compared to uninjected controls (n = 11). Control uninjected embryos displayed an overall average intensity of 13.2 while morphant embryos displayed an average intensity of 5.2. A one-way ANOVA was performed comparing intensity values of control uninjected to Stab2 MO injected embryos. These results show a role for Stab2 in regulating Erk phosphorylation in the dorsal aorta progenitors. These results correlate to an expansion of venous *stab1l* expression observed in seventy percent of Stab2 morphant embryos from the same experiment (Table S4).

**Figure 5:**
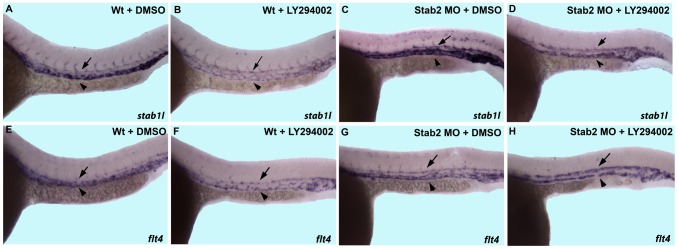
Downregulation of venous markers by PI3K inhibition is restored in Stab2 morphants. Both wild type and Stab2 morphant embryos were treated with either 1% DMSO or 20 µM LY294002 and stained for *stab1l* and *flt4* expression by in situ hybridization analysis. (A,B, E,F) Wild type embryos display a reduction in intensity of staining of either *stab1l* (A,B) or *flt4* (E,F) upon LY294002 treatment. Note that because venous *stab1l* and *flt4* expression is reduced, arterial and venous expressions appear of similar intensity. (C,G) DMSO treated Stab2 morphants display an expansion of *stab1l* (C) and *flt4* (G) expression into the area of the DA. (D,H) LY294002 treated Stab2 morphants display expanded arterial *stab1l* (D) and *flt4* (H) expression and the intensity of venous expression are restored to wild type levels. Arrows indicate DA and arrowheads indicated PCV. Lateral view of 24 hpf embryos, anterior is to the left, trunk and tail region is shown. Morphants were injected with a cocktail containing Stab2 MO1, MO2, and p53 MO.

As demonstrated previously, Vegf signaling regulates Erk phosphorylation by two opposing transduction pathways. The PI3 kinase branch inhibits PLC-γ signaling and Erk phosphorylation during A-V differentiation. Inhibition of PI3K function using the compound LY294002 resulted in increased P-Erk activation in zebrafish embryos and reduced venous differentiation [16]. We hypothesized that loss of Stab2 function would result in reduced P-Erk levels and restore venous differentiation in LY294002-treated embryos. Following Stab2 morpholino injection, embryos were treated with either 1% DMSO as a control, or 20 µM LY294002 starting at the 12-somite stage, and analyzed for venous *stab1l* and *flt4* expression at 24 hpf (Fig. 5). Wild type embryos treated with LY294002 displayed a reduced intensity of venous specific *stab1l* and *flt4* expression in contrast to DMSO treated controls (Fig. 5A,B,E,F, Table S4). Stab2 morphant embryos treated with DMSO displayed an expansion of *stab1l* and *flt4* venous expression as in previous experiments (Fig. 5C,G, Table S5). Stab2 morphants treated with LY294002 displayed increased venous *stab1l* and *flt4* staining as compared to control LY294002 treated embryos (Fig. 5D,H, Table S5). This argues that Stab2 inhibition reverses the reduced venous differentiation defects observed in LY294002 embryos. Similar to Stab2 morphants, Stab2 MO+LY294002 treated embryos exhibited increased *stab1l* and *flt4* expression in the DA and the absence of ISVs, therefore only partial rescue of the venous differentiation defects was observed.

### HA and Stab2 genetically interact in vivo

**Figure 6:**
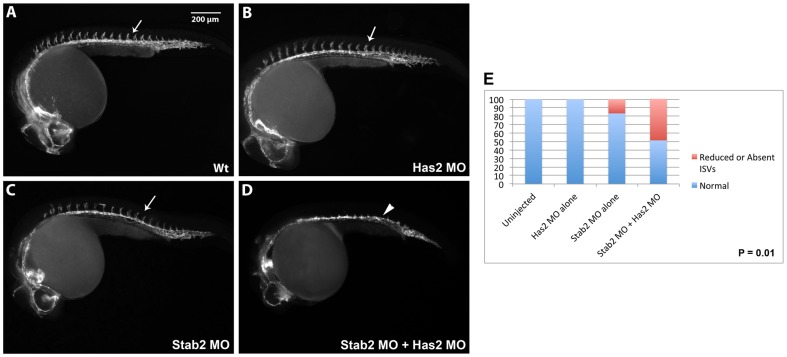
Stab2 and Has2 combinatorial knockdown results in a synergistic inhibitory effect on ISV formation. (A–C) A subphenotypic 1.25 ng dose of Stab2 (B) and 8 ng dose of Has2 (C) morpholinos do not affect vascular development when injected alone. (D) In contrast, Stab2 and Has2 MO co-injection results in 51 percent of embryos that lack ISVs suggesting that Stab2 and HA genetically interact. Arrows indicate ISVs. (E) Graph shows percentages of embryos displaying reduced or absent ISVs. Lateral view of 24 hpf embryos, anterior is to the left.

Given that human Stab2 has been previously shown to bind HA which resulted in P-Erk induction in vitro [23], we tested genetic interaction between zebrafish Stab2 and HA. Has2 is the only HA synthase expressed during early embryogenesis [36]. While it exhibits strongest expression in the paraxial mesoderm during somitogenesis stages [9], *has2* also shows significant expression of 28.9 reads per kb in *fli1a*:GFP-positive cells that include mostly vascular endothelial cells, based on our analysis of the recently published RNA-seq global expression study [37]. Has2 MO knockdown results in gastrulation defects due to HA requirement for gastrulation movements [36]; therefore it was not possible to analyze vascular differentiation in Has2 knockdown embryos. Thus, we co-injected low subphenotypic doses of Has2 MO and Stab2 MO to investigate if there is a synergistic interaction between HA and Stab2. We analyzed *kdrl*:GFP transgenic embryos for the absence of ISVs in both single and double morphant embryos. Both Has2 and Stab2 single morphants showed largely wild-type phenotype with only eighteen percent of Stab2 morphants and zero percent of Has2 morphants displaying a reduction in ISV growth (Fig. 6B,C,E, Table S6). In contrast, fifty-one percent of Has2 and Stab2 co-injected embryos displayed a severe reduction or loss of ISVs (Fig. 6D,E, Table S6). These results argue that Stab2 and HA genetically interact in vivo.

### Vegf but not Notch signaling affects *stab2* expression

**Figure 7:**
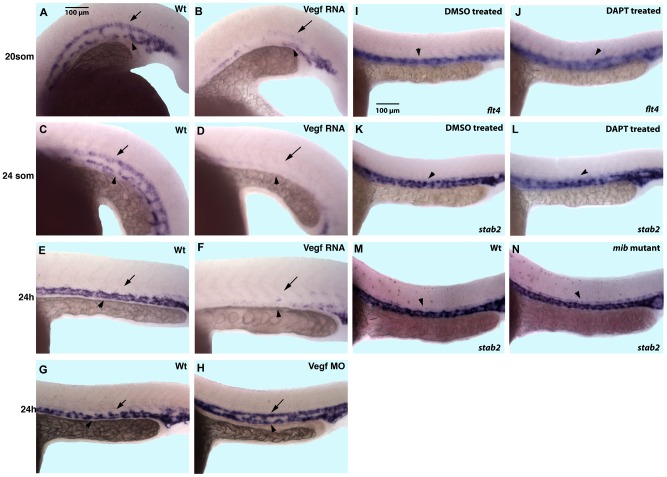
VEGF signaling inhibits *stab2* expression, while Notch inhibition has no effect on *stab2* expression. (A–F) Vegf overexpressing embryos display downregulated *stab2* expression evident by in situ hybridization analysis at the 20-somite (A,B), 24-somite (C,D) and 24 hpf (E,F) stages. Note that in wt embryos *stab2* is expressed in both the DA and the PCV at the 20–24-somite stage while its expression is primarily restricted to the PCV at 24 hpf. (G,H) Vegf morphants display an expansion of *stab2* expression into the DA at 24 hpf when expression is normally restricted to the PCV in wt embryos. (I–N) *Stab2* expression at 24 hpf is not affected by inhibition of Notch signaling either by DAPT treatment (K,L) or in *mindbomb* genetic mutants (M,N) while *flt4* expression is expanded in DAPT treated embryos (I,J). Arrows indicate DA and arrowheads indicate PCV in all panels. Lateral view, anterior is to the left, trunk and tail region is shown.

Vegf and Notch signaling have been previously implicated in A-V differentiation [12], [13]. To determine if Vegf signaling regulates Stab2 expression, we performed VegfA overexpression and knockdown by injecting RNA or morpholino respectively (Fig. 7). It has been previously reported that VegfA overexpression results in the expansion of arterial marker expression [12]. Embryos injected with a mixture of VegfA_121_ and VegfA_165_ RNA isoforms showed a reduction of venous *stab2* expression at 24 hpf (Fig. 7E,F). While *stab2* expression is localized to the PCV at 24 hpf, it is expressed in both arterial and venous precursors during 20–24-somite stages. Interestingly, both arterial and venous *stab2* expression was inhibited when Vegf RNA was overexpressed which indicates that Vegf can repress *stab2* expression both in arterial and venous progenitors (Fig. 7A–D). Vegf morphants showed an expansion of *stab2* expression into the DA at 24 hpf when expression is normally restricted to the PCV (Fig. 7G,H). This establishes that Vegf signaling inhibits *stab2* expression in the DA by 24 hpf during normal development, and helps to restrict it to the PCV.

To investigate if Notch signaling may regulate *stab2* expression, we knocked down Notch signaling using a DAPT chemical inhibitor (Fig. 7I–L) as well as examined *mindbomb* genetic mutants, deficient in Notch signaling [25], [26] (Fig. 7M,N). Neither DAPT treated embryos nor the genetic mutants showed any change in normal *stab2* expression by in situ hybridization analysis (arrowheads, Fig. 7K–N). In contrast, control venous *flt4* expression was expanded in DAPT-treated embryos as reported previously [11] (Fig. 7I–J). These results show that Vegf but not Notch signaling negatively regulates *stab2* expression.

## Discussion

These results demonstrate that Stabilin-2 function is required for arterial-venous differentiation in the major axial vessels during zebrafish embryogenesis. Stab2 knockdown results in a decrease in arterial Erk phosphorylation. We further demonstrate that downregulation of HA and Stab2 function results in a synergistic effect in support of the hypothesis that HA and Stab2 interact genetically. Expression of *stab2* itself is negatively regulated by Vegf signaling. These results argue that Stab2 participates in a signal transduction pathway in vivo and regulates A-V differentiation.

Previous studies have demonstrated that Stab2 functions as a receptor and physically interacts with HA [17], [18], [23]. Our data argue for genetic interaction of Stab2 and HA in vivo. Together these results support the hypothesis that Stab2 functions as a receptor for HA in a signal transduction pathway. However, the genetic synergy data presented do not exclude the possibility that Stab2 and HA in vivo may function in parallel pathways.

**Figure 8:**
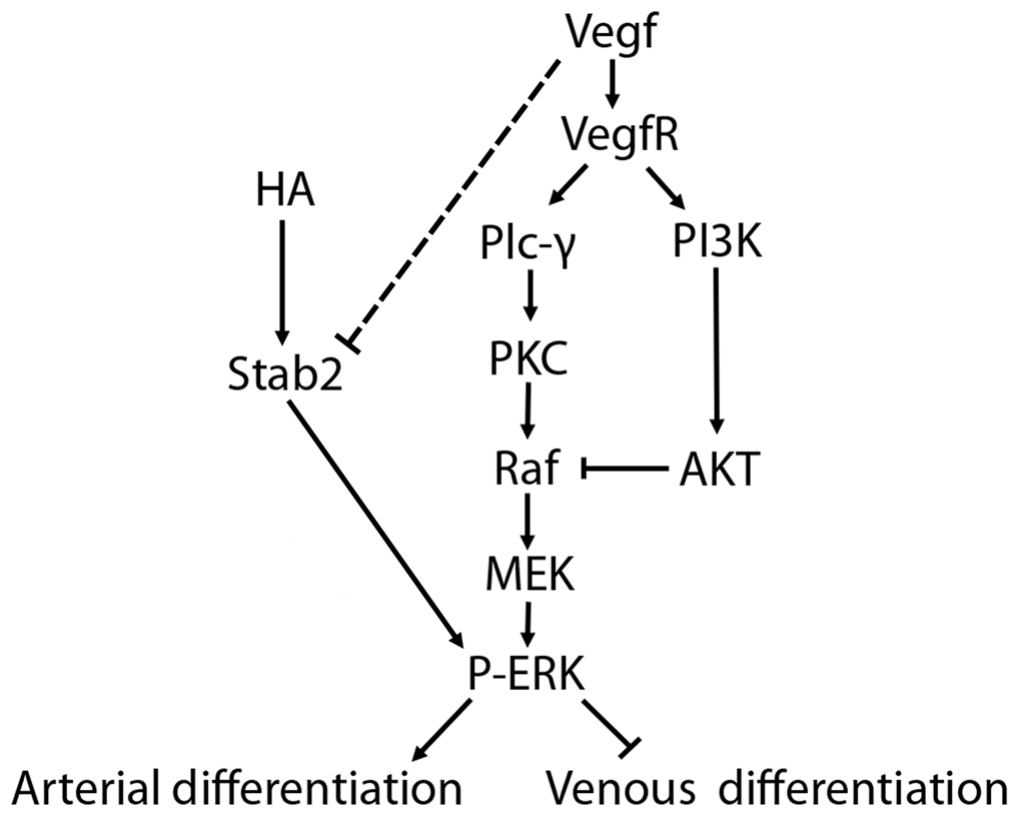
Stab2-HA pathway functions in parallel to the Vegf pathway. The proposed model shows that Stab2 interacts with HA to induce Erk phosphorylation, acting to upregulate arterial fate and suppressing venous fate in the arterial progenitors. This pathway may function in parallel to the Vegf pathway. Additionally, Vegf acts in a negative feedback loop to repress *stab2* expression, although the mechanism by which this occurs is unknown (indicated by dashed line).

Based on these results we suggest the following model for the role of Stab2 in A-V differentiation. We propose that HA-Stab2 signaling pathway functions in parallel to Vegf signaling to induce Erk phosphorylation in the arterial progenitors during mid-somitogenesis stages (Fig. 8). There also appears to be a regulatory mechanism whereby Vegf signaling also inhibits *stab2* transcription, aiding in restricting *stab2* expression to the cardinal vein by 24 hpf. As it has been previously shown, Erk phosphorylation promotes arterial and represses venous differentiation [2]. In support of this model, Erk phosphorylation is downregulated in a majority of Stab2 morphants. This also correlates with the expansion of venous marker expression observed in the absence of Stab2 function. Furthermore, loss of Stab2 function reverses the venous marker reduction phenotype, observed upon inhibition of PI3 kinase signaling in LY294002 treated embryos which, as demonstrated by the previous studies, results in increased Erk phosphorylation [2]. These results argue that the HA-Stab2 pathway functions in parallel to Vegf signaling to regulate Erk phosphorylation and A-V differentiation.

Stab2 exhibits a dynamic expression pattern and is first observed in the presumptive arterial progenitors while by 24 hpf its arterial expression is downregulated and venous expression is greatly increased. The observed A-V defects in Stab2 morphants are likely to be largely associated with early Stab2 expression because defects in ERK phosphorylation are observed as early as the 20-somite stage when Stab2 expression is mostly limited to the arterial progenitors. It is possible that venous Stab2 expression may have a different independent function such as involvement in lymphangiogenesis as it has been recently demonstrated [38]. As our results show, Vegf signaling can repress both arterial and venous *stab2* expression. However, during normal development Vegf signaling is only active in the arterial progenitors, therefore venous Stab2 expression is retained and enriched at 24 hpf. Why is *stab2* expression initiated in the arterial progenitors and not repressed by Vegf until the later stages? One possibility is that Vegf expression is weak during early stages of vasculogenesis. However, another explanation is that there is an autoregulatory loop where HA-Stab2 signaling serves to initiate Erk-phosphorylation, but after Erk has been phosphorylated, *stab2* expression is no longer required in the artery and is downregulated by P-Erk itself. Indeed, arterial *stab2* expression is retained in Stab2 morphants, which have reduced arterial P-Erk.

Knockdown of Stab2 results in a similar phenotype to knockdown of VegfA. Both VegfA and Stab2 morphants display a reduction in arterial P-Erk levels, as well as the expansion of venous markers. In the same experiments, similar percentages of Stab2 morphant embryos with expanded venous *stab1l* expression and decreased arterial P-Erk expression were observed. This correlation suggests that Stab2 morphants display expanded venous marker expression due to reduced Erk phosphorylation. Differently from VegfA morphants, arterial markers were not reduced in Stab2 morphants. In fact, a lesser fraction of Stab2 morphants showed an expansion of arterial marker *cldn5*. The cause of this phenotype is currently not clear. It is possible that P-Erk signaling is only partially downregulated in the arterial progenitors of Stab2 morphants, which is sufficient to allow for venous marker expression while insufficient to cause significant loss of arterial marker expression. Ectopic expression of arterial markers may be caused by defective migration of the first wave of endothelial precursors [4], which may become mislocalized when Erk phosphorylation pattern is perturbed.

Differently from zebrafish Stab2 knockdown embryos, mouse Stab2−/− mutants are viable [21]. While we cannot exclude the possibility that Stab2 has a different function in zebrafish than in mammalian embryos, it is also possible that mouse Stab1 and Stab2 may have partially redundant functions or A-V differentiation defects may potentially be more subtle and have not been examined in greater detail in mouse embryos. It is also possible that in mouse other pathways such as increased Vegf signaling may compensate for the loss of Stab2 and the phenotype may not appear until additional pathways are disturbed.

In summary, this study establishes the role for Stab2 signaling in arterial-venous differentiation. These results will promote our understanding of how arterial and venous fates are established during the development of the major axial vessels. It also raises an interesting possibility that Stab2 may potentially have additional signaling roles in the vascular system and its function is not limited to a scavenger receptor, as it has been previously believed.

## Supporting Information

Figure S1 **Two different Stab2 morpholinos result in similar phenotypes.** (A–D) In situ hybridization analysis shows *kdrl* RNA expression at 24 hpf in wild type uninjected (A), standard control morpholino injected (B) and embryos injected with two different Stab2 translation blocking morpholinos (C and D). *Kdrl* expression was unaffected by control morpholino injection. Stab2 morphants display a lack of ISVs. Embryos were microinjected with 3.75 ng of a standard control morpholino, 7.5 ng of Stab2 MO1, or 2.5 ng of Stab2 MO2. Arrows indicate ISVs (A and B) or missing ISVs (C and D).
(PDF)

Figure S2 **Specificity of Stab2 MO knockdown phenotype demonstrated by control 5 base pair mismatch Stab2 MO injection and a partial rescue by stab2 mRNA injection.** (A,D) Morphant embryos do not display any apparent reduction in ISVs by Tg(kdrl:GFP) expression at 24 hpf when injected with a 5 base pair mismatch Stab2 morpholino (Stab2 5 ms MO). (B,C,E,F) Stab2 5 ms MO-injected embryos show normal arterial cldn5a (B,E) and venous stab1l (C,F) expression. (G–I) Venous expansion of flt4 is apparent in Stab2 morphant embryos (H) as compared to uninjected controls (G), but is rescued by coinjection with stab2 mRNA (I). Lateral view of 24 hpf embryos shown, anterior is to the left. Morphants were injected with a cocktail containing 3.75 ng Stab2 5 ms MO+3.75 ng p53 MO (D–F), 3.75 ng Stab2 MO+3.75 ng p53 MO (H), or 3.75 ng Stab2 MO, 3.75 ng p53 MO and 160 pg stab2 mRNA (I). Arrows indicate DA and arrowheads indicate PCV in all panels.
(PDF)

Figure S3 **Stab2 morpholino disrupts Stab2 promoter driven GFP expression.** (A) Depiction of the stab2:GFP fusion construct created for injection. A 1 kb segment of stab2 upstream promoter/5′UTR sequence containing both morpholino sites (red and blue lines) and the translation start site (green line) was fused to the GFP coding region. (B) Embryos display mosaic GFP expression when injected with 40 pg of the stab2:GFP construct described in A. (B and C) This expression is downregulated when stab2 MO1 (C) or MO2 (D) are coinjected with stab2:GFP. Panels consist of a composite of representative embryos from each injection group at the 50% epiboly stage. (B) 29 out of 40 embryos express GFP. (C) 1 out of 23 embryos expressed GFP. (D) 1 out of 30 embryos expressed GFP.
(PDF)

Figure S4 **Translation of zebrafish** ***stab2*** **cDNA used for mRNA rescue.** DNA sequence has been modified to optimize codon usage and avoid MO binding. Protein sequence has not changed.
(PDF)

Table S1 **Stab2 knockdown results in a reduction in ISV expression as analyzed by whole mount ISH with 4 separate vascular endothelial markers.** Numbers and average percentages of embryos displaying absent or reduced ISVs when injected with a cocktail containing 3.75 ng total Stab2 MOs and 3.75 ng p53 MO. Value ± represents standard error. Analysis performed at the 24 hpf stage. All wild type uninjected embryos appeared normal.
(PDF)

Table S2 **Stab2 knockdown results in the expansion of several arterial and venous specific markers as analyzed with whole mount ISH analysis.** Numbers and average percentages of embryos displaying expansion when injected with a cocktail containing 3.75 ng total Stab2 MOs and 3.75 ng p53 MO. Value ± represents standard error. Analysis performed at 24 hpf. All uninjected embryos appeared normal.
(PDF)

Table S3 **Stab2 mRNA injection partially rescues the Stab2 morpholino knockdown phenotype.** Numbers and percentages of embryos displaying expanded *flt4* expression as analyzed by whole mount in situ hybridization when injected with either 3.75 ng of Stab2 MO+3.75 ng p53 MO or 3.75 ng Stab2 MO+3.75 ng p53 MO+160 pg *stab2* mRNA. Value ± represents standard error.
(PDF)

Table S4 **Stab2 knockdown results in decreased Erk phosphorylation and expanded venous marker expression.** Numbers and percentages of embryos displaying decreased Erk phosphorylation, as well as numbers and percentages of embryos from the same experiments displaying expanded expression of venous marker *stab1l* are shown. Value ± represents standard error. Embryos were injected with a cocktail containing 3.75 ng total Stab2 MOs and 3.75 ng p53 MO.
(PDF)

Table S5 **Inhibition of PI3K results in venous marker inhibition, but is restored in Stab2 morphants.** Numbers and percentages of LY294002 wild type embryos displaying reduced venous staining, Stab2 morphant untreated embryos displaying expanded venous marker expression, and Stab2 morphant LY294002 treated embryos displaying expanded venous marker expression. Value ± represents standard error. Stab2 Morphant embryos were injected with a cocktail containing 3.75 ng total Stab2 MOs and 3.75 ng p53 MO. Embryos were treated with 20 µM LY294002.
(PDF)

Table S6 **Subphenotyic doses of Stab2 MO+Has2 MO result in a synergistic effect as analyzed in Tg(** ***kdrl*** **:GFP embryos).** Numbers and percentages of embryos displaying absent or reduced ISVs when injected with 1.25 ng Stab2 MO+1.25 ng p53 MO and/or 8 ng Has2 MO+2.5 ng p53 MO. Value ± represents standard error. Embryos were then analyzed for missing or severely reduced ISV expression at the 24 hpf stage.
(PDF)
