# Supplementary material for: Hyaluronic Acid Receptor Stabilin-2 Regulates Erk Phosphorylation and Arterial - Venous Differentiation in Zebrafish
Source: PLoS One. 2014 Feb 28;9(2):e88614. doi: 10.1371/journal.pone.0088614 (PMC3938420; doi:10.1371/journal.pone.0088614)
Supplement: Figure S1 — Two different Stab2 morpholinos result in similar phenotypes. (A–D) In situ hybridization analysis shows kdrl RNA expression at 24 hpf in wild type uninjected (A), standard control morpholino injected (B) and embryos injected with two different Stab2 translation blocking morpholinos (C and D). Kdrl expression was unaffected by control morpholino injection. Stab2 morphants display a lack of ISVs. Embryos were microinjected with 3.75 ng of a standard control morpholino, 7.5 ng of Stab2 MO1, or 2.5 ng of Stab2 MO2. Arrows indicate ISVs (A and B) or missing ISVs (C and D). (PDF) [file pone.0088614.s001.pdf]

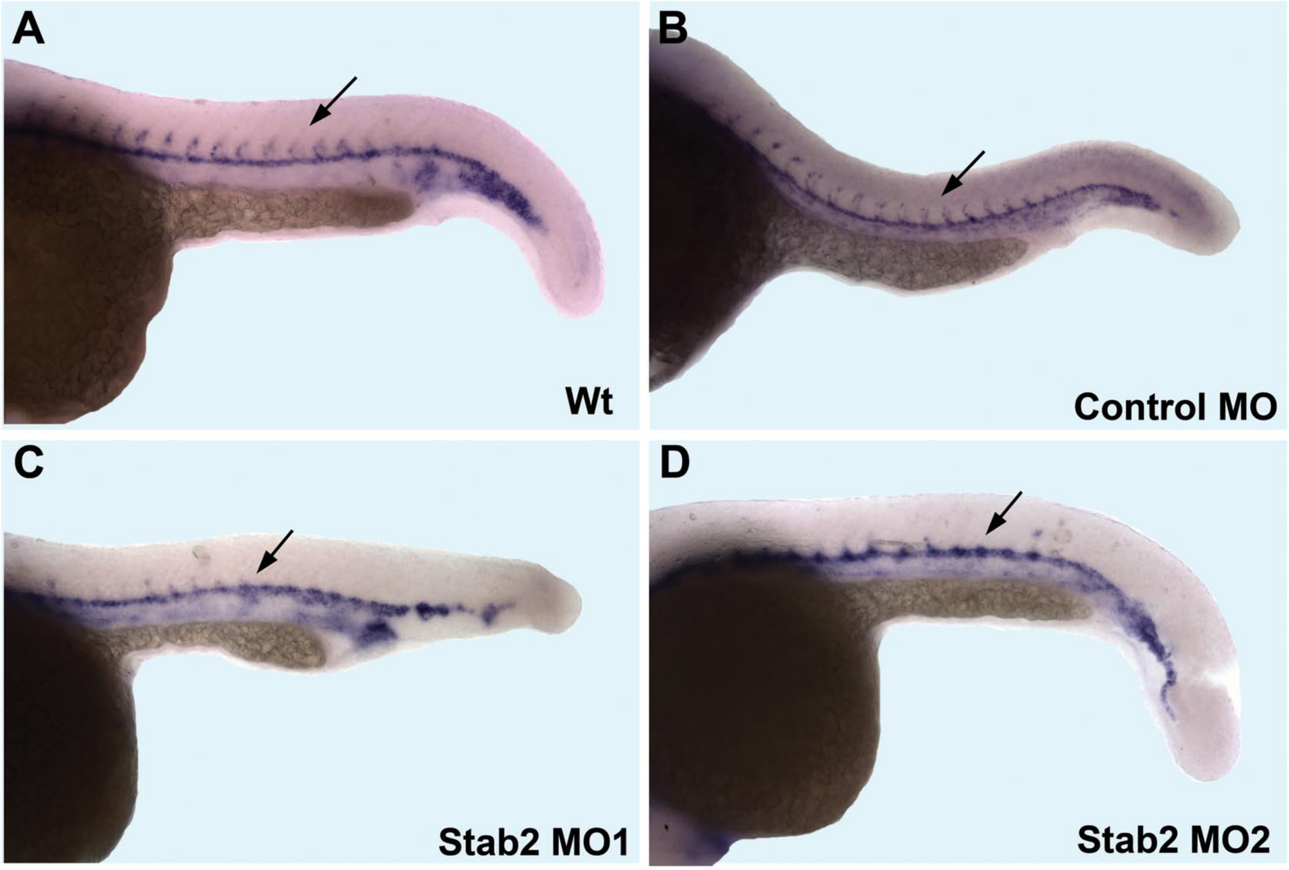

Suppl. Figure S1: Two different Stab2 morpholinos result in similar phenotypes. (A-D) In situ hybridization analysis shows *kdrl* RNA expression at 24 hpf in wild type uninjected (A), standard control morpholino injected (B) and embryos injected with two different Stab2 translation blocking morpholinos (C and D). *Kdrl* expression was unaffected by control morpholino injection. Stab2 morphants display a lack of ISVs. Embryos were microinjected with 3.75 ng of a standard control morpholino, 7.5 ng of Stab2 MO1, or 2.5 ng of Stab2 MO2. Arrows indicate ISVs (A and B) or missing ISVs (C and D).
