# Supplementary material for: Hyaluronic Acid Receptor Stabilin-2 Regulates Erk Phosphorylation and Arterial - Venous Differentiation in Zebrafish
Source: PLoS One. 2014 Feb 28;9(2):e88614. doi: 10.1371/journal.pone.0088614 (PMC3938420; doi:10.1371/journal.pone.0088614)
Supplement: Figure S2 — Specificity of Stab2 MO knockdown phenotype demonstrated by control 5 base pair mismatch Stab2 MO injection and a partial rescue by stab2 mRNA injection. (A,D) Morphant embryos do not display any apparent reduction in ISVs by Tg(kdrl:GFP) expression at 24 hpf when injected with a 5 base pair mismatch Stab2 morpholino (Stab2 5 ms MO). (B,C,E,F) Stab2 5 ms MO-injected embryos show normal arterial cldn5a (B,E) and venous stab1l (C,F) expression. (G–I) Venous expansion of flt4 is apparent in Stab2 morphant embryos (H) as compared to uninjected controls (G), but is rescued by coinjection with stab2 mRNA (I). Lateral view of 24 hpf embryos shown, anterior is to the left. Morphants were injected with a cocktail containing 3.75 ng Stab2 5 ms MO+3.75 ng p53 MO (D–F), 3.75 ng Stab2 MO+3.75 ng p53 MO (H), or 3.75 ng Stab2 MO, 3.75 ng p53 MO and 160 pg stab2 mRNA (I). Arrows indicate DA and arrowheads indicate PCV in all panels. (PDF) [file pone.0088614.s002.pdf]

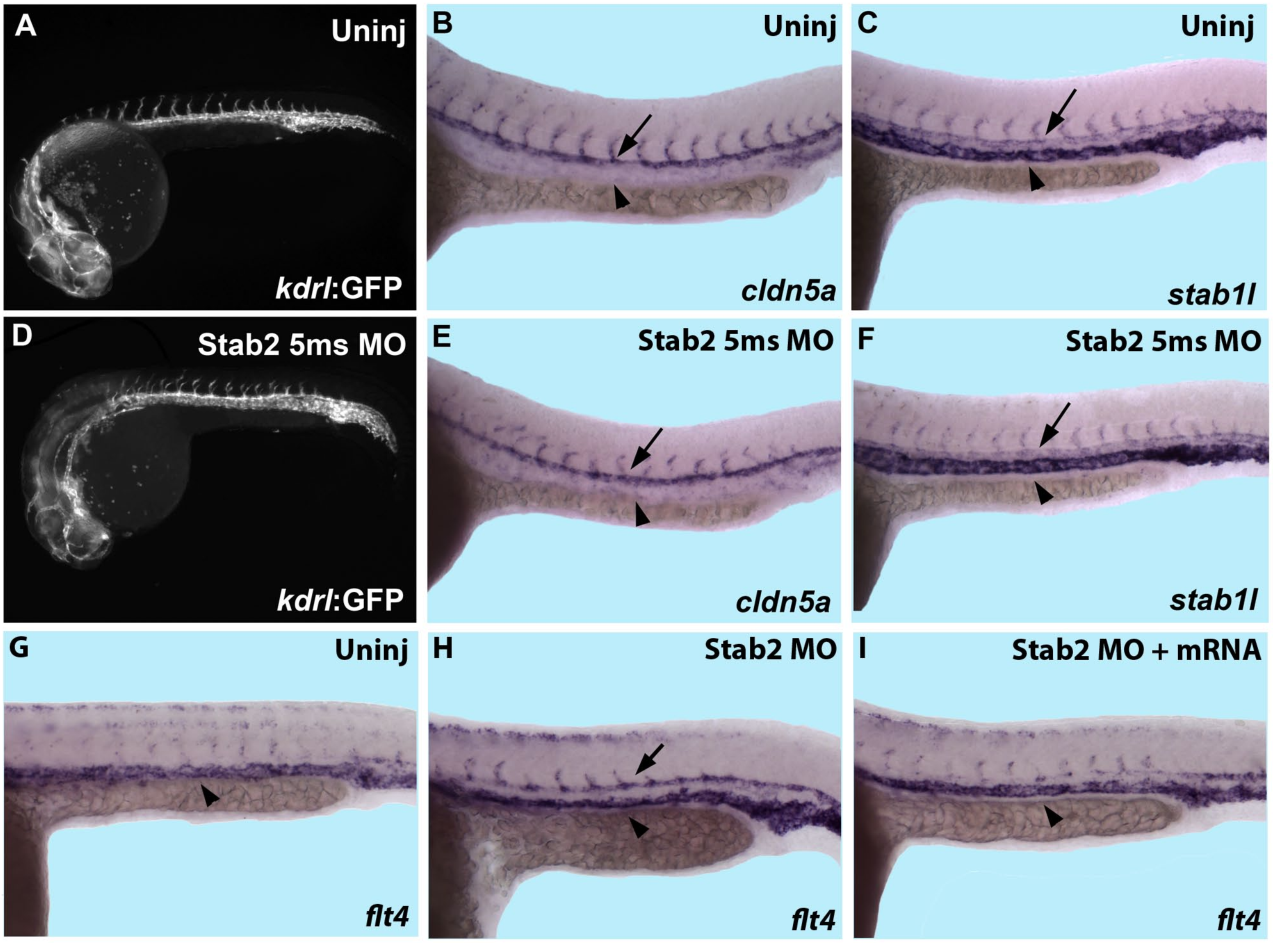

Supplemental Figure S2. Specificity of Stab2 MO knockdown phenotype demonstrated by control 5 base pair mismatch Stab2 MO injection and a partial rescue by *stab2* mRNA injection. (A,D) Morphant embryos do not display any apparent reduction in ISVs by Tg(*kdrl:*GFP) expression at 24 hpf when injected with a 5 base pair mismatch Stab2 morpholino (Stab2 5ms MO). (B,C,E,F) Stab2 5 ms MO-injected embryos show normal arterial *cldn5a* (B,E) and venous *stab1l* (C,F) expression. (G-I) Venous expansion of *flt4* is apparent in Stab2 morphant embryos (H) as compared to uninjected controls (G), but is rescued by coinjection with *stab2* mRNA (I). Lateral view of 24 hpf embryos shown, anterior is to the left. Morphants were injected with a cocktail containing 3.75 ng Stab2 5ms MO + 3.75 ng p53 MO (D-F), 3.75 ng Stab2 MO + 3.75 ng p53 MO (H), or 3.75 ng Stab2 MO, 3.75 ng p53 MO and 160 pg *stab2* mRNA (I). Arrows indicate DA and arrowheads indicate PCV in all panels.
