# Supplementary material for: Hyaluronic Acid Receptor Stabilin-2 Regulates Erk Phosphorylation and Arterial - Venous Differentiation in Zebrafish
Source: PLoS One. 2014 Feb 28;9(2):e88614. doi: 10.1371/journal.pone.0088614 (PMC3938420; doi:10.1371/journal.pone.0088614)
Supplement: Figure S3 — Stab2 morpholino disrupts Stab2 promoter driven GFP expression. (A) Depiction of the stab2:GFP fusion construct created for injection. A 1 kb segment of stab2 upstream promoter/5′UTR sequence containing both morpholino sites (red and blue lines) and the translation start site (green line) was fused to the GFP coding region. (B) Embryos display mosaic GFP expression when injected with 40 pg of the stab2:GFP construct described in A. (B and C) This expression is downregulated when stab2 MO1 (C) or MO2 (D) are coinjected with stab2:GFP. Panels consist of a composite of representative embryos from each injection group at the 50% epiboly stage. (B) 29 out of 40 embryos express GFP. (C) 1 out of 23 embryos expressed GFP. (D) 1 out of 30 embryos expressed GFP. (PDF) [file pone.0088614.s003.pdf]

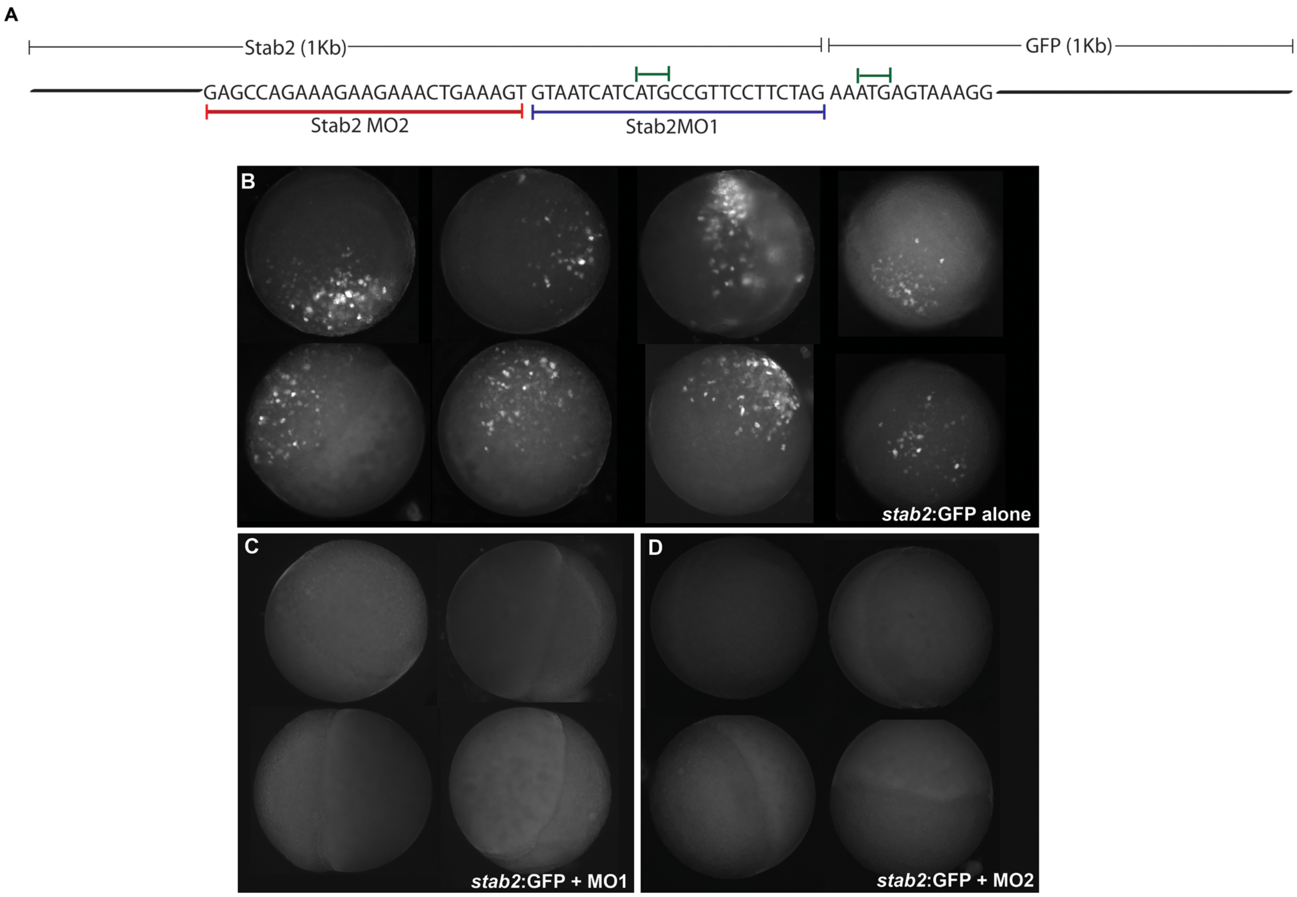

Suppl. Figure S3. Stab2 morpholino disrupts Stab2 promoter driven GFP expression. *(A)* Depiction of the *stab2*:GFP fusion construct created for injection. A 1kb segment of *stab2* upstream promoter / 5'UTR sequence containing both morpholino sites (red and blue lines) and the translation start site (green line) was fused to the GFP coding region. (B) Embryos display mosaic GFP expression when injected with 40pg of the *stab2*:GFP construct described in A. (B and C) This expression is downregulated when stab2 MO1 (C) or MO2 (D) are coinjected with *stab2*:GFP. Panels consist of a composite of representative embryos from each injection group at the 50% epiboly stage. (B) 29 out of 40 embryos express GFP. (C) 1 out of 23 embryos expressed GFP. (D) 1 out of 30 embryos expressed GFP.
