# Supplementary material for: Hyaluronic Acid Receptor Stabilin-2 Regulates Erk Phosphorylation and Arterial - Venous Differentiation in Zebrafish
Source: PLoS One. 2014 Feb 28;9(2):e88614. doi: 10.1371/journal.pone.0088614 (PMC3938420; doi:10.1371/journal.pone.0088614)
Supplement: Figure S4 — Translation of zebrafish stab2 cDNA used for mRNA rescue. DNA sequence has been modified to optimize codon usage and avoid MO binding. Protein sequence has not changed. (PDF) [file pone.0088614.s004.pdf]

```
1/1                                        31/11
|                                          |
ATG CCC TTC CTG CTG GAC GTG GTG TGC GGC GGA CTG ATC CTG CTG CTG GGA ACC GAA ATC
M   P   F   L   L   D   V   V   C   G   G   L   I   L   L   L   G   T   E   I

61/21                                      91/31
|                                          |
CTG TGG GTC GCT GGC GCT CAG AAA CGC TGC GAC AAA GAT ATC ACA ATG GTG ACC AAG AGC
L   W   V   A   G   A   Q   K   R   C   D   K   D   I   T   M   V   T   K   S

121/41                                     151/51
|                                          |
AAA TGT CAC AGT TGC AGC CTG TCT CAT CTG GCT AAA TGC CCT AAC GGA TAC GCC AAG ATG
K   C   H   S   C   S   L   S   H   L   A   K   C   P   N   G   Y   A   K   M

181/61                                     211/71
|                                          |
CCT CCA GGC ACT AGC GAC AGG GAT TGT CGC TAC TAT GTG ACA CCC GCA TTC TCC ATC TCA
P   P   G   T   S   D   R   D   C   R   Y   Y   V   T   P   A   F   S   I   S

241/81                                     271/91
|                                          |
GGA TGT TCT TTC AAC TGC TTT AAG GAG GTG GTC GAA CCT CAG TGT TGC AGG GGT TAT TGG
G   C   S   F   N   C   F   K   E   V   V   E   P   Q   C   C   R   G   Y   W

301/101                                    331/111
|                                          |
GGA AAT GAT TGT ATG GAG TGC CCT GGC AGT GCT AGC ACC CCA TGT TCA AAC AAT GGT GTG
G   N   D   C   M   E   C   P   G   S   A   S   T   P   C   S   N   N   G   V

361/121                                    391/131
|                                          |
TGC AGT GAC GGA ATC GCT GGT AAC GGA ACA TGT ACC TGC GCT TCT GGC TTC ACT GGT GCT
C   S   D   G   I   A   G   N   G   T   C   T   C   A   S   G   F   T   G   A

421/141                                    451/151
|                                          |
GCC TGT GAG GAA TGC AAA ACC GAC CTG TAC GGA CCA ACT TGC TCC AAC GTG TGT CGC TGC
A   C   E   E   C   K   T   D   L   Y   G   P   T   C   S   N   V   C   R   C

481/161                                    511/171
|                                          |
AAA AAT GGC CTG TGT AGC TCT GGT CTG AAG GGC ACT GGA GAG TGT ACA TGC TTT TCT GGA
K   N   G   L   C   S   S   G   L   K   G   T   G   E   C   T   C   F   S   G

541/181                                    571/191
|                                          |
TAT ACA GGC CTG GAT TGC GCC CAG GAA CTG CCC GCA TGT GCA GCT CTG CAG TGC GGA CCT
Y   T   G   L   D   C   A   Q   E   L   P   A   C   A   A   L   Q   C   G   P

601/201                                    631/211
|                                          |
GAC TCC AGA TGT ATC GAG GAA ATG CTG ACT GGT CAG CTG GTG TGT AAG TGC AAA CCT GGA
D   S   R   C   I   E   E   M   L   T   G   Q   L   V   C   K   C   K   P   G

661/221                                    691/231
|                                          |
TAC CAG GGA GAT GGC GTC CAG TGC ACC TCT ATT AAC CCA TGT CTG AGG TCC GTC TGC CAC
Y   Q   G   D   G   V   Q   C   T   S   I   N   P   C   L   R   S   V   C   H
```

```
721/241                             751/251
|                                   |
GCT AAC GCC GTG TGT GCT CAC ACT GGA CCT AAT AAA CAT GTG TGT ACT TGC ACA GAG GGC
A   N   A   V   C   A   H   T   G   P   N   K   H   V   C   T   C   T   E   G

781/261                             811/271
|                                   |
TAC AGC GGT GAT GGA AGG GTC TGT ATG CCT ATC GAC CCA TGC CAG ACT AAC CTG GGA AAT
Y   S   G   D   G   R   V   C   M   P   I   D   P   C   Q   T   N   L   G   N

841/281                             871/291
|                                   |
TGC ACC TCT GGC TCC ACT CGC TGT GTG TAT GAC GGC CCA GGT AAA GCT CAC TGT GAG TGC
C   T   S   G   S   T   R   C   V   Y   D   G   P   G   K   A   H   C   E   C

901/301                             931/311
|                                   |
CTG AAA GGA TTC GAG AAG TTT GTC GAA GGA CAG GGC TGC TCT ATC ATT GAC CTG TGT AAA
L   K   G   F   E   K   F   V   E   G   Q   G   C   S   I   I   D   L   C   K

961/321                             991/331
|                                   |
CCC GAT TCC TGC CAT AAG TAC GCC ACT TGT GCA ACA GCT GAA CCT GGC ACC GTG GAG TGT
P   D   S   C   H   K   Y   A   T   C   A   T   A   E   P   G   T   V   E   C

1021/341                            1051/351
|                                   |
AAC TGC AGA GAA GGT TAT ATC GGT AAT GGA AAG ATT TGT TTC GGA GAT ATC ATT CAG CAG
N   C   R   E   G   Y   I   G   N   G   K   I   C   F   G   D   I   I   Q   Q

1081/361                            1111/371
|                                   |
CTG AAC GAG ATG AAT TCT AAA CCT GGA GGC AAA TGG ACA GGC CAG CTG TCC TCA GCC ATC
L   N   E   M   N   S   K   P   G   G   K   W   T   G   Q   L   S   S   A   I

1141/381                            1171/391
|                                   |
ACC CTG TTC AAC CAA GTG ACT GGT TGG TTT CTG ACA GCA AGG GGA CCC TTC ACT GTC TTT
T   L   F   N   Q   V   T   G   W   F   L   T   A   R   G   P   F   T   V   F

1201/401                            1231/411
|                                   |
GTG CCT GTC AAT AAG GCT TTT AAA GGA ACA AGT GTG AAA AGC CTG CTG GCC GAC GAG ATG
V   P   V   N   K   A   F   K   G   T   S   V   K   S   L   L   A   D   E   M

1261/421                            1291/431
|                                   |
AAA GCC CGC TAC CTG GCA AAG CTG CAC GTG GTC GCT GGA GAG GTG AAC TCA GAA AGT CTG
K   A   R   Y   L   A   K   L   H   V   V   A   G   E   V   N   S   E   S   L

1321/441                            1351/451
|                                   |
AAG AAA GGC ATC CTG TTC CAT ACC CTG ACT GGC ATG GGT GCT GAG TCA ATG ATC GAT GCC
K   K   G   I   L   F   H   T   L   T   G   M   G   A   E   S   M   I   D   A

1381/461                            1411/471
|                                   |
GAA CAG GTG AAG ATT CGC CTG CAC GGT AGT AGA AAG AAA GGA GCC CTG GTC GAG TCC GAC
E   Q   V   K   I   R   L   H   G   S   R   K   K   G   A   L   V   E   S   D

1441/481                            1471/491
|                                   |
ATT TTT GCA TCA AAC GGA ATG ATC CAT CTG ATT GAC AAA CTG ATG GAT GCT GTG CCT AGT
I   F   A   S   N   G   M   I   H   L   I   D   K   L   M   D   A   V   P   S
```

1501/**501** 1531/**511**

ACA GTC ATC AGC GAG AAG GAG GAA AGC CTG CTG GAA ATT CTG TCT AAG AAC GGA AAA TTC
T V I S E K E E S L L E I L S K N G K F

1561/**521** 1591/**531**

TCT CAG TTT AAA TCC CTG CTG GAG AAG ACA AAT GTG GCA ACC GTC CTG GAG GAA GAT GGC
S Q F K S L L E K T N V A T V L E E D G

1621/**541** 1651/**551**

CCA TAC ACC CTG TTC GCT CCC ACT AAC GTG GCC TTT GCA CTG ATG AAA CCA GAC TAC CTG
P Y T L F A P T N V A F A L M K P D Y L

1681/**561** 1711/**571**

GCC TAT CTG AAG AGC GAG GAA GGA AAG ACC AAA CTG CTG GAG CTG ATG AGA AAC CAC ATC
A Y L K S E E G K T K L L E L M R N H I

1741/**581** 1771/**591**

GTG GCA ACA ACC CCC CTG TCC GCT TAT GTG ATT GTC TCA AAT CCT AGG GCT GTG ACA ATG
V A T T P L S A Y V I V S N P R A V T M

1801/**601** 1831/**611**

GCT GAG CAG ATC CTG ACT TTC AAC GTC ACT ACA GCC GGT CAG ATC CTG GTG AAT GGA GAG
A E Q I L T F N V T T A G Q I L V N G E

1861/**621** 1891/**631**

TCT GTC ATT GAA CTG GAT GTG GAG GCA AAA AAC GGC CGC CTG CAC TCA CTG GAG GGT CTG
S V I E L D V E A K N G R L H S L E G L

1921/**641** 1951/**651**

CTG ATC CCC CCT AGT ATC GAA CCC ATT CTG CCT CAT AGA TGC GAC ATC ATT AAG CCT ATC
L I P P S I E P I L P H R C D I I K P I

1981/**661** 2011/**671**

AAA TAC ATG GCT CCA TGT GTG AGC TGC ACC CTG ATT AGC AAA TCT ACC TGC CCC ACT GGA
K Y M A P C V S C T L I S K S T C P T G

2041/**681** 2071/**691**

GAG TCT CTG AAG GCC TAT AGA AGG GGC TGT GTG TTT AAC AAG AAA CAG CTG GGA CTG TCT
E S L K A Y R R G C V F N K K Q L G L S

2101/**701** 2131/**711**

CTG CCT TCC ATC GGC TGT TCT CAC ATT TGC AAT GAT ACT AAA ACC ACT CCA CAG TGT TGC
L P S I G C S H I C N D T K T T P Q C C

2161/**721** 2191/**731**

AAG GGT TTC TTT GGA CCC GAC TGT TCA CCC TGC CCT GGT GGA TTC ACA ACC CCC TGT AGT
K G F F G P D C S P C P G G F T T P C S

2221/**741** 2251/**751**

AGC CAC GGA ACC TGC AGT GAG GGT ATC GAC GGA AAC GGC ACT TGT CAG TGC GAA CCT AAG
S H G T C S E G I D G N G T C Q C E P K

2281/**761** 2311/**771**
TTT AAA GGA AGC AGA TGT CAG TAC TGC GCA GAT TCC AAC AAA TAT GGC CCT AAT TGC GAC
F K G S R C Q Y C A D S N K Y G P N C D

2341/**781** 2371/**791**
AAG ACA TGT TGG TGC ATC CAC GGT ACC TGT GAT AAC CAT CCA GAG GCT TCT GGA AAG TGT
K T C W C I H G T C D N H P E A S G K C

2401/**801** 2431/**811**
AAA CAG GGC AGT TGC AAG GAC GGC TAC ACT GGA GAG TAT TGT GAA CTG CAG ACA CAG CCA
K Q G S C K D G Y T G E Y C E L Q T Q P

2461/**821** 2491/**831**
TGT GGA CCC AAT CAG CCT TGC CAC GCT CAT GCC AAC TGT GTC TCC AAT AAA GGT GCT TTC
C G P N Q P C H A H A N C V S N K G A F

2521/**841** 2551/**851**
ACA TGT GTG TGC AAG CCT GGA TTT CAG GGT GAT GGA TAC ATG TGC ATG GAG TCT GAC CCT
T C V C K P G F Q G D G Y M C M E S D P

2581/**861** 2611/**871**
TGT GCA CTG CCA CAC AGA GGC GGT TGC TCC AAA AAC GCT ATC TGT ATT AAG ACT GGC CCA
C A L P H R G G C S K N A I C I K T G P

2641/**881** 2671/**891**
GGT ACA CAT AAG TGT AAA TGC CTG AGT GGT TGG CGC GAA GAC GGA GAT GAG TGC CAG GCC
G T H K C K C L S G W R E D G D E C Q A

2701/**901** 2731/**911**
ATC AAC AAT TGT CTG GAC CCT AGC AGA GGA GGC TGC CAC CCA AAT GCC ACT TGT ATC TAC
I N N C L D P S R G G C H P N A T C I Y

2761/**921** 2791/**931**
GTG GGA CCA GGC CAG ATT GAC TGT GCA TGC AAA AGC GGA TAT CAT GGT AAC GGA AGG GAG
V G P G Q I D C A C K S G Y H G N G R E

2821/**941** 2851/**951**
TGC GAA CCC GTG AAT CAG TGT GTC GAG CAG AAG GGT GGA TGC CAC TTC CTG GCT ACA TGT
C E P V N Q C V E Q K G G C H F L A T C

2881/**961** 2911/**971**
CAG TTT CTG AAC CCT GAT GGA TGG CAT TGT GTG TGC GAG GAC GGC TAC GCC GGC GAT GGT
Q F L N P D G W H C V C E D G Y A G D G

2941/**981** 2971/**991**
AAA ATC TGC TAT GGC ACT CTG CTG CAG GAG GTC TCC ACA AAC CCC GAC CTG CTG GGA TTC
K I C Y G T L L Q E V S T N P D L L G F

3001/**1001** 3031/**1011**
AAT CAG TGG ATT TCA AAG GCT GAG CTG TCA CAG CTG CTG AGT GAG AGA GAA AAC CTG ACT
N Q W I S K A E L S Q L L S E R E N L T

3061/**1021** 3091/**1031**

CTG TTC GTG CCT TCA GCA CAG GCT ATC GAG AAG ATG AGT AAA GAA GAC AAG GAT TTT TGG
L F V P S A Q A I E K M S K E D K D F W

3121/**1041** 3151/**1051**

ATG ACA CCC AGC AAC CTG CCT TCT CTG GTG AAA AAT CAC ATT CTG TCC GGA TCA TTT ACA
M T P S N L P S L V K N H I L S G S F T

3181/**1061** 3211/**1071**

CTG AGC GAT CTG AAT ACC AGT CCA AGC CCA CCC CCT AGA CTG GTC AGC CTG CTG AAG AGG
L S D L N T S P S P P P R L V S L L K R

3241/**1081** 3271/**1091**

ACT CTG CCC GTG TAC TCT ACA AAC GAC ACT ACA ATC GTC GCC GGC GGT AAA ATT ACC TTC
T L P V Y S T N D T T I V A G G K I T F

3301/**1101** 3331/**1111**

GGT GAT ATG GCC GCA AAG AAT GGA GTC ATC CAC CTG ATT GAC ACT GTG CTG ATC CCT GAG
G D M A A K N G V I H L I D T V L I P E

3361/**1121** 3391/**1131**

CAG CAG ATG TCC GAA GGC CTG CTG CAG GTG CTG AAG AAG AGA AGC GAC CTG AGT CTG TTT
Q Q M S E G L L Q V L K K R S D L S L F

3421/**1141** 3451/**1151**

TAT GGA AGT CTG CTG AAC CAT AAT CTG ACA AAC GAG ATG GAG GAA AGC CTG GGC TTC ACA
Y G S L L N H N L T N E M E E S L G F T

3481/**1161** 3511/**1171**

GTG TTT GCT CCA ACC GAC AAT GCC ATC CAG GAT TAC CTG AAA AGA ACC GGA AAG GAG AGC
V F A P T D N A I Q D Y L K R T G K E S

3541/**1181** 3571/**1191**

CTG GAC CTG AAC GTG ACC ATG TAT CAC ATC ATT CTG ACT GAA ATT CTG AAA GAC GTG GAT
L D L N V T M Y H I I L T E I L K D V D

3601/**1201** 3631/**1211**

CTG GTC GAC GGC CTG TAC AAG GAT ACA ATG CTG GGC TTC CAG TAT CAG CTG GGT TTC TTT
L V D G L Y K D T M L G F Q Y Q L G F F

3661/**1221** 3691/**1231**

CGC CAG GAT AAA AGA CTG CTG GTG AAC GAG GCT GAA GTG AAT GTC ACC GAC CTG GAG ACT
R Q D K R L L V N E A E V N V T D L E T

3721/**1241** 3751/**1251**

AGC AAG GGA GTC ATC CAC ATC ATT AGC GCC GTG CTG AAC ATT CCA TCT AAT AGG TGT GAT
S K G V I H I I S A V L N I P S N R C D

3781/**1261** 3811/**1271**

AAA GCA ACC TCC ATC CTG TTC TCA GGA CGC TGT ATG GAC TGC TTT CAG ACA ATC ATT AAC
K A T S I L F S G R C M D C F Q T I I N

3841/**1281**   3871/**1291**
| |
CCA TGC CCC TCC GGC ACC AAG AAA GTG ATG TCA AGG AAG CGC AGA TGT ATG TAC TCT CGC
P   C   P   S   G   T   K   K   V   M   S   R   K   R   R   C   M   Y   S   R

3901/**1301**   3931/**1311**
| |
GTC CTG CAC GGC GAT ACA CTG CTG ACC ATC GGT TGT AAA TTT TCA TGC GAG AAG ATG ACA
V   L   H   G   D   T   L   L   T   I   G   C   K   F   S   C   E   K   M   T

3961/**1321**   3991/**1331**
| |
ATC GAA AGA ATT TGT TGC AGT GGA CAT TAT GGC ATT AAC TGT GAG AAA TGC CCT GGC AGT
I   E   R   I   C   C   S   G   H   Y   G   I   N   C   E   K   C   P   G   S

4021/**1341**   4051/**1351**
| |
GAA GAT CAG AGC TGC TTC GGA AAC GGC ATC TGT AAG GAC GGA ATT AAT GGT ACC GGA GTG
E   D   Q   S   C   F   G   N   G   I   C   K   D   G   I   N   G   T   G   V

4081/**1361**   4111/**1371**
| |
TGT CTG TGC AAC CAG GGT TTT AAT GGA ACT GCC TGT GAG AGC TGC CAG CCA GGC AAA TAC
C   L   C   N   Q   G   F   N   G   T   A   C   E   S   C   Q   P   G   K   Y

4141/**1381**   4171/**1391**
| |
GGT ATC CAC TGC GAC CAG GAA TGT AAA TGC GTG CAT GGC AGG TGT AAG GAT GGT ATT GAC
G   I   H   C   D   Q   E   C   K   C   V   H   G   R   C   K   D   G   I   D

4201/**1401**   4231/**1411**
| |
GGC GAT GGT TCC TGT GCT TGC GAC CTG GGA TGG AGG GGT GTG AAC TGC GAT ATC GCC ATT
G   D   G   S   C   A   C   D   L   G   W   R   G   V   N   C   D   I   A   I

4261/**1421**   4291/**1431**
| |
AAA TCA GAC ATG TGT GGT GGC AAG TGC CAC TCT TCC GCC AAC TGT CTG GTG AAT GTG GTC
K   S   D   M   C   G   G   K   C   H   S   S   A   N   C   L   V   N   V   V

4321/**1441**   4351/**1451**
| |
GAT TCA AGT TAC TAT TGT TCA TGC GCT GCC GGA TTC CAG GGA AAC GGC ACC TAC TGC GAG
D   S   S   Y   Y   C   S   C   A   A   G   F   Q   G   N   G   T   Y   C   E

4381/**1461**   4411/**1471**
| |
GCA ATC GAC GCC TGT GCA GAA AGA AAT GGT GGA TGC AGC GCT CAC GCC GTG TGT AAA AGG
A   I   D   A   C   A   E   R   N   G   G   C   S   A   H   A   V   C   K   R

4441/**1481**   4471/**1491**
| |
ACA CTG CCT GGC AGG CGC ATC TGT ATG TGC CAT CCA GGT TAT GAG GGT GAC GGA AAG GTG
T   L   P   G   R   R   I   C   M   C   H   P   G   Y   E   G   D   G   K   V

4501/**1501**   4531/**1511**
| |
TGC ACC AGT ATT AAC CCT TGT CTG GAT GGC GTC AAT GGC GGT TGC CAC GCA GAC AGC AAC
C   T   S   I   N   P   C   L   D   G   V   N   G   G   C   H   A   D   S   N

4561/**1521**   4591/**1531**
| |
TGT ATC CAT ACT GGA CCA AAT AAA ACA GCT TGT CAC TGC AAG GAG GGA TTT TAC AAA GAT
C   I   H   T   G   P   N   K   T   A   C   H   C   K   E   G   F   Y   K   D

4621/**1541** 4651/**1551**
GGC AAG AAC TGC GAG CTG ATC AAT CTG TGT AAG GAG AAG AAC GGA GGC TGC CAC CGC GCA
G K N C E L I N L C K E K N G G C H R A

4681/**1561** 4711/**1571**
GCT ATT TGT ACA ATG ACC GGT CCT CTG CAG AGA AAT TGT ACA TGC CAT GCT GGA TAC GTG
A I C T M T G P L Q R N C T C H A G Y V

4741/**1581** 4771/**1591**
GGC GAC GGA GAG ATT TGT AAG TAT ACC CTG CAG AGA GAA CTG CCA ATT GGA ACT GCC AGG
G D G E I C K Y T L Q R E L P I G T A R

4801/**1601** 4831/**1611**
ACA GCA TTC GTG AAA GCA ATG TTT AAG AAC CGC ATC TAC GAG CTG GAT ACC AGA GGC CCT
T A F V K A M F K N R I Y E L D T R G P

4861/**1621** 4891/**1631**
TTC ACT GTC TTC GCT TTC AGA AGT GAA AAC TAC AAG AAC CAG AGC ATT GAC CTG CTG AAG
F T V F A F R S E N Y K N Q S I D L L K

4921/**1641** 4951/**1651**
AGG ACA CCA GAG AAT ACC AGA ATC ATG AGG TAC CAC ATT GTG TGT TGC AGA ACA CTG CTG
R T P E N T R I M R Y H I V C C R T L L

4981/**1661** 5011/**1671**
CCC CAG GAT CTG ATG CAG CCT AGG AAC CTG ACC ACT CTG AGC GGA GAC ATC CTG TCT ATT
P Q D L M Q P R N L T T L S G D I L S I

5041/**1681** 5071/**1691**
ACA TAC TCC GAG AAC ACC ATC TAC ATC AAC AAC AAG GCC AAG GTG CTG TTT TCC GAT ATC
T Y S E N T I Y I N N K A K V L F S D I

5101/**1701** 5131/**1711**
GAG AGC TCT AAC GGC ATC CTG CAT GAA ATT GAC TCA GTG CTG GTC CCA CCC GGA CTG GAG
E S S N G I L H E I D S V L V P P G L E

5161/**1721** 5191/**1731**
ATT CGC GAA GAC AAG AAA CAG GAT ATC ATT CCT AGA AAC TTC TCA GAA GTG GCA AGT GCT
I R E D K K Q D I I P R N F S E V A S A

5221/**1741** 5251/**1751**
AAC GGA TTC AAG ACC TTC TTT AAG CTG CTG GAG GAC ACT GAT ACA CTG AAA CTG GTC ATG
N G F K T F F K L L E D T D T L K L V M

5281/**1761** 5311/**1771**
GAC CCA ATC CAC CAG CCC GTG ACT CTG TTC ATG CCA ACA GAC GAT GCT ATG AGC GCC CTG
D P I H Q P V T L F M P T D D A M S A L

5341/**1781** 5371/**1791**
TCT CAG GAA CAG AAA GAT TTT CTG TAC GCC ATG CAC AAC AGA GAC AAG CTG TCT GAG TAC
S Q E Q K D F L Y A M H N R D K L S E Y

5401/**1801** 5431/**1811**

CTG AGG TAT CAT ATC CTG CGC GAT ACT AAA CTG ATG GCC TCC GAG CTG ATT TAC GCA TCC
L R Y H I L R D T K L M A S E L I Y A S

5461/**1821** 5491/**1831**

TCA CTG AAG ACA CAG CAG GGA AGC GAC CTG TCA GTG GCA TGC ATG GGA GAG GAA CAG ATC
S L K T Q Q G S D L S V A C M G E E Q I

5521/**1841** 5551/**1851**

GGA GAG CTG TAT GTC AAC CAC AAA GGA TGT AGG ATT GTG AAG CGC TAC CTG GAT TTC AAC
G E L Y V N H K G C R I V K R Y L D F N

5581/**1861** 5611/**1871**

GGT GGA ATG ATC TAT GGC ATT GAC TGC CTG CTG AAT CCT CCA TCC CTG GGC GGT AGA TGT
G G M I Y G I D C L L N P P S L G G R C

5641/**1881** 5671/**1891**

GAT CAT AGG CAG ACT ATC GAC TTT ACA CTG GCC TGT AGA AGC TGC GGA AGT AGC ATT ATG
D H R Q T I D F T L A C R S C G S S I M

5701/**1901** 5731/**1911**

GAT TGC CCA CTG GGC TCT AAG CCT GGA GGA GAG CAG GAA TGT GTC CTG CCA GAC AGT GTG
D C P L G S K P G G E Q E C V L P D S V

5761/**1921** 5791/**1931**

ATC AGC AGA GGA TCT AAA TGT CAG TCT ACA TGC AAG GTG GTC GTG TGG AAG CCC AAA TGT
I S R G S K C Q S T C K V V V W K P K C

5821/**1941** 5851/**1951**

TGC TCC GGT TAC TAT GGA AGG GAT TGT CTG GCA TGC CCA GGT GGA CCC CAG TCC CCT TGC
C S G Y Y G R D C L A C P G G P Q S P C

5881/**1961** 5911/**1971**

TCA AAC CAC GGC AAA TGT GAC GAG GAT CAT CTG GGA AAT GGC ACC TGT ACT TGC GAC ACC
S N H G K C D E D H L G N G T C T C D T

5941/**1981** 5971/**1991**

GGC TTC ACT GGT GTG GCT TGT GAG TCT TGC CTG GAT GGA CAC TTT GGC CCT GAC TGC AAG
G F T G V A C E S C L D G H F G P D C K

6001/**2001** 6031/**2011**

GCT TGT AAC TGC ACA GAG CAT GGC TCC TGT GAT GAA GGT CTG CAG GGT ACA GGA TCA TGT
A C N C T E H G S C D E G L Q G T G S C

6061/**2021** 6091/**2031**

TTC TGC GAG GAA GGT TGG ACC GGA CCA CAG TGC GAG AAC AAA CTG GCA GAT GGA CCC GTG
F C E E G W T G P Q C E N K L A D G P V

6121/**2041** 6151/**2051**

TGT AAG TAC TGG AAT GGC GGT TGC AGT AAA GAC GCT AAG TGT AGC CAG AAA GGA GAG AAA
C K Y W N G G C S K D A K C S Q K G E K

6181/**2061** 6211/**2071**

GTG AGC TGT GCC TGC CTG CAG GGA TTC TCT GGC GAC GGT TTT GTC TGC ACC CCA ATC GAC
V S C A C L Q G F S G D G F V C T P I D

6241/**2081** 6271/**2091**

CCC TGT GTG GAT GGA GAG AAC GGA GGC TGC CAC GAA CAT GCT ACA TGT ACC ATG ACT GGA
P C V D G E N G G C H E H A T C T M T G

6301/**2101** 6331/**2111**

CCA GGC AAG CGC AAG TGT GAG TGC AAA GAT TCT TAC GTG GGT GAC GGA ATC GAT TGC GAA
P G K R K C E C K D S Y V G D G I D C E

6361/**2121** 6391/**2131**

GCC AAG GTG CTG CCC GTC AAC AGA TGT CTG GTC GAC AAT GGA CAG TGC CAC TCA GAC GCA
A K V L P V N R C L V D N G Q C H S D A

6421/**2141** 6451/**2151**

CAG TGT ACC GAT CTG CAC CAT GAG GAC AAA ACT CTG GGT GTG TTC CAT TAT AGA AGT ACA
Q C T D L H H E D K T L G V F H Y R S T

6481/**2161** 6511/**2171**

AAC GGA ACC TAC AAA CTG AAT TAT ACT ATG GCA CAG GAA GCT TGT AAG GAG GCA GGC AGC
N G T Y K L N Y T M A Q E A C K E A G S

6541/**2181** 6571/**2191**

ACT ATC GCT ACA TAC ATG CAG CTG TCC TAT GCC CAG CAG GCA GGA TAC TCA CTG TGC ACA
T I A T Y M Q L S Y A Q Q A G Y S L C T

6601/**2201** 6631/**2211**

GCT GGC TGG CTG GAT AAA GCT CGC GTG GCC TAT CCA ATG AGT TTC TCT TCC CCC AGA TGT
A G W L D K A R V A Y P M S F S S P R C

6661/**2221** 6691/**2231**

GGC AGC GGT CAC GTG GGA ATC GTC GAC TAC GGC GTG AGG AAC AAT CTG AGC GAG ACC TGG
G S G H V G I V D Y G V R N N L S E T W

6721/**2241** 6751/**2251**

GAT ACT TTT TGC TAC CGC GTG AAG GAC GTC CAG TGT AAG TGC AAA ACA GGC TAT ATC GGA
D T F C Y R V K D V Q C K C K T G Y I G

6781/**2261** 6811/**2271**

GAT GGC TTC AGT TGT ATT GGT AAC CTG ATG CAG GTG CTG AGC GCC ACA CCC ACC CTG AGC
D G F S C I G N L M Q V L S A T P T L S

6841/**2281** 6871/**2291**

AAC TTT CTG TCT CAG ATC CTG AAT TAC TCT ACC TCC GCA TCA GGA AAG GCT TTC GTG AAC
N F L S Q I L N Y S T S A S G K A F V N

6901/**2301** 6931/**2311**

AGA CTG AGG AAT ATC ACC ATT CAG TCT ACT CTG TTT GTC CCA GAC AAC GAT GGC CTG TAT
R L R N I T I Q S T L F V P D N D G L Y

```
6961/2321                                         6991/2331
|                                                 |
AGT AAT CAG ACT CTG AGC GGT AGG GAT ATC GAG CAC CAT CTG CTG GAC GGA AGG GCA CTG
S   N   Q   T   L   S   G   R   D   I   E   H   H   L   L   D   G   R   A   L

7021/2341                                         7051/2351
|                                                 |
ATC CTG CAG GCA CTG ATT AAC GTG ACA CAC GTC CGC ACC AGA CTG GGT TCA AGT CTG ACT
I   L   Q   A   L   I   N   V   T   H   V   R   T   R   L   G   S   S   L   T

7081/2361                                         7111/2371
|                                                 |
ATT ACA GGA GTG CCA GAC CTG CAG AAC CCC CAG AAA ATG ACA AGC TCT GGC TAC ATC AAT
I   T   G   V   P   D   L   Q   N   P   Q   K   M   T   S   S   G   Y   I   N

7141/2381                                         7171/2391
|                                                 |
GAT AGA TAT GTG ATC GAC TCT AAC ATT TTC GCT TCC AAT GGC GTG ATT CAT GTC CTG CAG
D   R   Y   V   I   D   S   N   I   F   A   S   N   G   V   I   H   V   L   Q

7201/2401                                         7231/2411
|                                                 |
GGT CCT CTG AAG GCA CCA CCT CCA CCA ACC CCT TCC TTT CAC CCA GCA CAT CAG GCT GGT
G   P   L   K   A   P   P   P   P   T   P   S   F   H   P   A   H   Q   A   G

7261/2421                                         7291/2431
|                                                 |
CTG GGA ATC GGC GTG CTG GGA CTG ATC ATT CTG ATT GTC GTG GCT GGC TTC GTC GGT TAC
L   G   I   G   V   L   G   L   I   I   L   I   V   V   A   G   F   V   G   Y

7321/2441                                         7351/2451
|                                                 |
AAC TTT TAT ACA CAC AAG ACC AAA CCA TTC CAG TTT TAC TAT TTC AAA GGA GAC GAG GGC
N   F   Y   T   H   K   T   K   P   F   Q   F   Y   Y   F   K   G   D   E   G

7381/2461                                         7411/2471
|                                                 |
GAA GAT GTG ACC CCT ACT GAG TCC TCA CCA AAC ATC AGC AAT CCA GTC TAC GAC GCA GCA
E   D   V   T   P   T   E   S   S   P   N   I   S   N   P   V   Y   D   A   A

7441/2481                                         7471/2491
|                                                 |
CCA GCA ACC AAG GAG CAG CCT CCA ACC GAG GAG GAC AAA CAT CAG GTC ATC TCA AGT GGC
P   A   T   K   E   Q   P   P   T   E   E   D   K   H   Q   V   I   S   S   G

7501/2501
|
GTC TTC GAG CTG CTG CAG GAC TCA TGA
V   F   E   L   L   Q   D   S   *
```

Supplemental Figure S4. Translation of zebrafish *stab2* cDNA used for mRNA rescue. DNA sequence has been modified to optimize codon usage and avoid MO binding. Protein sequence has not changed.
