# Supplementary material for: Hyaluronic Acid Receptor Stabilin-2 Regulates Erk Phosphorylation and Arterial - Venous Differentiation in Zebrafish
Source: PLoS One. 2014 Feb 28;9(2):e88614. doi: 10.1371/journal.pone.0088614 (PMC3938420; doi:10.1371/journal.pone.0088614)
Supplement: Table S1 — Stab2 knockdown results in a reduction in ISV expression as analyzed by whole mount ISH with 4 separate vascular endothelial markers. Numbers and average percentages of embryos displaying absent or reduced ISVs when injected with a cocktail containing 3.75 ng total Stab2 MOs and 3.75 ng p53 MO. Value ± represents standard error. Analysis performed at the 24 hpf stage. All wild type uninjected embryos appeared normal. (PDF) [file pone.0088614.s005.pdf]

	Total N counted	Percent with reduced or absent ISVs
fli1a	25	56 ± 1.3
kdrl	31	77 ± 8.9
esam	31	48 ± 1.7
she	30	63 ± 0.9

Suppl. Table S1. Stab2 knockdown results in a reduction in ISV expression as analyzed by whole mount ISH with 4 separate vascular endothelial markers. Numbers and average percentages of embryos displaying absent or reduced ISVs when injected with a cocktail containing 3.75 ng total Stab2 MOs and 3.75 ng p53 MO. Value ± represents standard error. Analysis performed at the 24 hpf stage. All wild type uninjected embryos appeared normal.
