# Supplementary material for: Hyaluronic Acid Receptor Stabilin-2 Regulates Erk Phosphorylation and Arterial - Venous Differentiation in Zebrafish
Source: PLoS One. 2014 Feb 28;9(2):e88614. doi: 10.1371/journal.pone.0088614 (PMC3938420; doi:10.1371/journal.pone.0088614)
Supplement: Table S2 — Stab2 knockdown results in the expansion of several arterial and venous specific markers as analyzed with whole mount ISH analysis. Numbers and average percentages of embryos displaying expansion when injected with a cocktail containing 3.75 ng total Stab2 MOs and 3.75 ng p53 MO. Value ± represents standard error. Analysis performed at 24 hpf. All uninjected embryos appeared normal. (PDF) [file pone.0088614.s006.pdf]

	Total N Counted	Percent with expanded expression
aqp8	37	49 ± 7.0
cldn5b	96	43 ± 14.3
grl	34	0 ± 0.0
flt4	35	66 ± 6.7
mrc1	63	38 ± 5.7
stab1l	70	73 ± 8.3
stab2	44	77 ± 21.7

Suppl. Table S2. Stab2 knockdown results in the expansion of several arterial and venous specific markers as analyzed with whole mount ISH analysis. Numbers and average percentages of embryos displaying expansion when injected with a cocktail containing 3.75 ng total Stab2 MOs and 3.75 ng p53 MO. Value ± represents standard error. Analysis performed at 24 hpf. All uninjected embryos appeared normal.
