# Supplementary material for: Hyaluronic Acid Receptor Stabilin-2 Regulates Erk Phosphorylation and Arterial - Venous Differentiation in Zebrafish
Source: PLoS One. 2014 Feb 28;9(2):e88614. doi: 10.1371/journal.pone.0088614 (PMC3938420; doi:10.1371/journal.pone.0088614)
Supplement: Table S3 — Stab2 mRNA injection partially rescues the Stab2 morpholino knockdown phenotype. Numbers and percentages of embryos displaying expanded flt4 expression as analyzed by whole mount in situ hybridization when injected with either 3.75 ng of Stab2 MO+3.75 ng p53 MO or 3.75 ng Stab2 MO+3.75 ng p53 MO+160 pg stab2 mRNA. Value ± represents standard error. (PDF) [file pone.0088614.s007.pdf]

	Total N counted	Percentage with expanded expression
Stab2 MO	31	90 ± 5
Stab2 MO+mRNA	31	52 ± 1

Suppl. Table S3. Stab2 mRNA injection partially rescues the Stab2 morpholino knockdown phenotype. Numbers and percentages of embryos displaying expanded *flt4* expression as analyzed by whole mount in situ hybridization when injected with either 3.75 ng of Stab2 MO + 3.75 ng p53 MO or 3.75 ng Stab2 MO + 3.75 ng p53 MO + 160 pg *stab2* mRNA. Value ± represents standard error.
