# Supplementary material for: Hyaluronic Acid Receptor Stabilin-2 Regulates Erk Phosphorylation and Arterial - Venous Differentiation in Zebrafish
Source: PLoS One. 2014 Feb 28;9(2):e88614. doi: 10.1371/journal.pone.0088614 (PMC3938420; doi:10.1371/journal.pone.0088614)
Supplement: Table S4 — Stab2 knockdown results in decreased Erk phosphorylation and expanded venous marker expression. Numbers and percentages of embryos displaying decreased Erk phosphorylation, as well as numbers and percentages of embryos from the same experiments displaying expanded expression of venous marker stab1l are shown. Value ± represents standard error. Embryos were injected with a cocktail containing 3.75 ng total Stab2 MOs and 3.75 ng p53 MO. (PDF) [file pone.0088614.s008.pdf]

| | Control Uninjected | Stab2 MO |
|---|---|---|
| Decreased arterial P-Erk expression | 14.4% ± 6<br>N = 28 | 73.5% ± 5<br>N = 34 |
| Increased *stab1l* expression | 0.0% ± 0<br>N = 37 | 70% ± 1<br>N = 37 |

**Suppl. Table S4. Stab2 knockdown results in decreased Erk phosphorylation and expanded venous marker expression.** Numbers and percentages of embryos displaying decreased Erk phosphorylation, as well as numbers and percentages of embryos from the same experiments displaying expanded expression of venous marker *stab1l* are shown. Value ± represents standard error. Embryos were injected with a cocktail containing 3.75 ng total Stab2 MOs and 3.75 ng p53 MO
