# Supplementary material for: Hyaluronic Acid Receptor Stabilin-2 Regulates Erk Phosphorylation and Arterial - Venous Differentiation in Zebrafish
Source: PLoS One. 2014 Feb 28;9(2):e88614. doi: 10.1371/journal.pone.0088614 (PMC3938420; doi:10.1371/journal.pone.0088614)
Supplement: Table S5 — Inhibition of PI3K results in venous marker inhibition, but is restored in Stab2 morphants. Numbers and percentages of LY294002 wild type embryos displaying reduced venous staining, Stab2 morphant untreated embryos displaying expanded venous marker expression, and Stab2 morphant LY294002 treated embryos displaying expanded venous marker expression. Value ± represents standard error. Stab2 Morphant embryos were injected with a cocktail containing 3.75 ng total Stab2 MOs and 3.75 ng p53 MO. Embryos were treated with 20 µM LY294002. (PDF) [file pone.0088614.s009.pdf]

| | Wt LY294002 treated embryos Total N | Percentage Showing lighter expression | Stab2 MO embryos Total N | Percentage Showing expanded expression | Stab2 MO + LY294002 treatment embryos total N | Percentage showing expanded but not lighter expression |
|---|---|---|---|---|---|---|
| stab1l | 42 | 62 ± 27.7 | 33 | 67 ± 20.6 | 39 | 87 ± 13.1 |
| flt4 | 39 | 49 ± 6.7 | 30 | 57 ± 5.5 | 32 | 88 ± 16.7 |

**Suppl. Table S5. Inhibition of PI3K results in venous marker inhibition, but is restored in Stab2 morphants.** Numbers and percentages of LY294002 wild type embryos displaying reduced venous staining, Stab2 morphant untreated embryos displaying expanded venous marker expression, and Stab2 morphant LY294002 treated embryos displaying expanded venous marker expression. Value ± represents standard error. Stab2 Morphant embryos were injected with a cocktail containing 3.75 ng total Stab2 MOs and 3.75 ng p53 MO. Embryos were treated with 20 µM LY294002.
