# Supplementary material for: Hyaluronic Acid Receptor Stabilin-2 Regulates Erk Phosphorylation and Arterial - Venous Differentiation in Zebrafish
Source: PLoS One. 2014 Feb 28;9(2):e88614. doi: 10.1371/journal.pone.0088614 (PMC3938420; doi:10.1371/journal.pone.0088614)
Supplement: Table S6 — Subphenotyic doses of Stab2 MO+Has2 MO result in a synergistic effect as analyzed in Tg( kdrl :GFP embryos). Numbers and percentages of embryos displaying absent or reduced ISVs when injected with 1.25 ng Stab2 MO+1.25 ng p53 MO and/or 8 ng Has2 MO+2.5 ng p53 MO. Value ± represents standard error. Embryos were then analyzed for missing or severely reduced ISV expression at the 24 hpf stage. (PDF) [file pone.0088614.s010.pdf]

	Total N counted	Percent with reduced or absent ISVs
wt	58	0 ± 0
Stab2 MO	62	18 ± 3.8
Has2 MO	63	0 ± 0
Stab2 MO + Has2 MO	55	51 ± 0.7

Suppl. Table S6. Subphenotyic doses of Stab2 MO + Has2 MO result in a synergistic effect as analyzed in Tg(*kdrl*:GFP embryos). Numbers and percentages of embryos displaying absent or reduced ISVs when injected with 1.25 ng Stab2 MO + 1.25 ng p53 MO and/or 8ng Has2 MO + 2.5 ng p53 MO. Value ± represents standard error. Embryos were then analyzed for missing or severely reduced ISV expression at the 24 hpf stage.
